# The Iconic Atlantic Goliath Grouper (*Epinephelus itajara*): A Comprehensive Assessment of Health Indices in the Southeastern United States Population

**DOI:** 10.3389/fvets.2020.00635

**Published:** 2020-09-25

**Authors:** Christopher R. Malinowski, Justin R. Perrault, Felicia C. Coleman, Christopher C. Koenig, Justin M. Stilwell, Carolyn Cray, Nicole I. Stacy

**Affiliations:** ^1^Florida State University Coastal and Marine Laboratory, St. Teresa, FL, United States; ^2^Department of Biological Science, Florida State University, Tallahassee, FL, United States; ^3^Department of Forestry and Natural Resources, Purdue University, West Lafayette, IN, United States; ^4^Mote Marine Laboratory, Sarasota, FL, United States; ^5^Loggerhead Marinelife Center, Juno Beach, FL, United States; ^6^Department of Pathology, College of Veterinary Medicine, University of Georgia, Athens, GA, United States; ^7^Division of Comparative Pathology, Department of Pathology & Laboratory Medicine, University of Miami Miller School of Medicine, Miami, FL, United States; ^8^Department of Comparative, Diagnostic, and Population Medicine, College of Veterinary Medicine, University of Florida, Gainesville, FL, United States

**Keywords:** hematology, histology, marine teleost, native immune functions, oxidative stress, plasma biochemistry, conservation ecology

## Abstract

The Atlantic Goliath Grouper (*Epinephelus itajara*) population has rebounded from near extinction to an international status as vulnerable due in part to regional species recovery efforts. The southeastern US population has been recovering with the main spawning locations off the coasts of Florida. Despite their economic importance to the catch-and-release fishery and the dive industry, and their ecological importance as ecosystem engineers resulting in positive impacts on reefs and species richness, baseline health assessment information is very limited in this species to date. The objectives of this study were to: (1) establish reference intervals for hematological and plasma biochemical analytes, and report immune function, oxidative stress, and vitellogenin in mature males and females; (2) evaluate total length, age, and sex in relation to blood analytes in juvenile and mature fish; (3) assess analytes across sampled months in mature male and female fish; and (4) describe the typical light microscopy findings in liver and gill biopsies, including quantitative assessment of pigmented macrophage aggregates. Health indices are reported as reference intervals when applicable, or otherwise descriptively. Blood analyte correlations with length and age, sex differences, and comparisons across months provided relevant physiological considerations, including differences in protein/energy metabolism, tissue growth, sexual maturation, active reproduction, and antigenic stimulation. Liver histology identified changes associated with life stage, active reproduction, or of subclinically to clinically insignificant infectious and/or inflammatory processes. Hepatocellular vacuolation and pigmented macrophage aggregates were prominent. Pigmented macrophage aggregates correlated with total length, presumably from continuous antigenic stimulation and/or metabolic changes as fish grow. Gill histological findings were subtle. The data presented herein provide an essential baseline assessment of a suite of health variables in an iconic marine teleost species, serves as a springboard for future studies relevant to conservation physiology, and allows for population-level applications for conservation management and policy.

## Introduction

Conservation physiology is a newly emerging discipline that examines the health status and physiological responses of wildlife to environmental disturbances in an effort to better understand factors contributing to population declines (1, 2). These assessments are crucial for conservation efforts, as wildlife are increasingly faced with such anthropogenic threats as climate change, habitat loss, pathogen introduction, contaminant exposure, and overexploitation (3–5). Many of these perturbations are occurring at unprecedented rates and have already resulted in recognizable declines in individual and population health, making conservation and recovery efforts challenging (4, 5). Population-level health assessments involve a combination of physical and physiological examinations in an effort to establish “baselines” for hematological and biochemical analytes (2–4). It is when shifts in these biomarkers occur that physiologically relevant alterations in population health in response to various stressors can be recognized (6, 7). Population health and viability are inseparable, and therefore monitoring the health of organisms and their populations can be used to inform management and to improve upon our understanding of complex conservation issues (2, 3).

Comprehensive health assessments of threatened and endangered wildlife species are much rarer in comparison to domestic and aquaculture species (8–10). Hematological and biochemical analyses can be useful in identifying stress, inflammation, diseases, and nutritional deficiencies, among many other underlying conditions or responses to physiological changes. However, these diagnostic procedures are less frequently utilized in fishes compared to other vertebrate species, whether mammalian or not (9, 10). Baseline health studies in fishes have primarily focused on aquaculture species, including salmon [*Salmo salar*, (11)], tilapia [*Oreochromis* sp., (12)], sturgeon [*Acipenser* spp., (13, 14)], and seabass [*Dicentrarchus labrax*, (9)]. Reports of hematological and biochemical blood analytes are much less common for wild teleosts and elasmobranchs (15–19). Because of the paucity of information on baseline blood analytes in wild fishes, it is crucial to establish reference intervals in marine fish populations to better understand overall health and disease prevalence. These reference intervals can then be used as a basis for investigating spatial and temporal trends and for assessing population dynamics after potential environmental changes, stressors, or specific threats that might occur over time (5).

One internationally vulnerable marine fish species is the Atlantic Goliath Grouper (*Epinephelus itajara*; hereafter, called Goliath Grouper) (20). This species is the largest grouper in the Atlantic (reaching adult sizes of >2 m total length and 360–450 kg), long-lived (at least 37 years), charismatic, and it resides in waters of the western Atlantic Ocean, Gulf of Mexico, and Caribbean south to Brazil (21–24). Goliath Grouper are characterized as slow-maturing, protogynous hermaphrodites (21, 25) with known spawning locations in coastal waters of Florida in the southeastern United States (26, 27) and off the southern coast of Brazil (28). Largely due to overfishing, these animals faced near extinction in the early 1990s. They now show signs of population recovery off the coasts of Florida due to the closing of commercial and recreational fisheries from extractive exploitation but remain in low numbers elsewhere throughout their range. Several factors continue to cast doubt on whether full recovery has occurred and will be possible in Florida, including loss of mangrove habitat, destructive episodic events such as red tide and sudden cold exposure in winter months, and health and reproductive effects of high mercury concentrations in tissues and gonads (29–32). Due to conservation efforts in the United States, Florida now serves as the center of recovery for the remaining Goliath Grouper population (32, 33).

Baseline blood analyses can be used to assess sublethal impacts of environmental contaminants, but it is necessary to first establish reference intervals to gauge what is “physiologically normal” in these animals (34). Such assessments can, and should, be conducted using non-lethal methods whenever possible. Therefore, the objectives of this study were to use non-lethal sampling methods (1) to establish reference intervals for hematological and plasma biochemical analytes, and report immune function, oxidative stress, and vitellogenin in mature males and females; (2) to evaluate total length, age, and sex in relation to blood analytes in juvenile and mature fish; (3) to assess analytes across sampled months in mature male and female fish; and (4) describe the typical light microscopy findings in liver and gill biopsies, including quantitative assessment of pigmented macrophage aggregates.

## Materials and Methods

### Capture, Tissue Sampling and Preparation

This research was performed in accordance with institutional and national guidelines concerning the use of animals in research, and was approved by the Florida State University Institutional Animal Care and Use Committee (IACUC) (protocol #s: 1106, 1411, and 1718); Florida Fish and Wildlife Conservation Commission permits SAL-15-1244A-SRP, SAL-16-1244A-SRP, SAL-17-1244-SRP; and the National Oceanic and Atmospheric Administration permit F/SER24:PH.

This work was conducted throughout the coastal waters of Florida [for capture and sampling region details see (31)] at depths between 10 and 35 m for adults and <0.1–2 m for juveniles. Juveniles were distinguished from mature adults based on sampling location, size, and age. Juveniles primarily occur in mangrove habitat, while adults occur on offshore reefs. Males mature at 1,100–1,150 mm total length and 4–6 yrs of age, while females mature at 1,200–1,350 mm total length and 6–7 yrs of age (21, 35).

Several of us (26, 31, 35) developed non-lethal capture and sampling efforts that addressed federal and state laws requiring the release of this protected species alive and in good condition. On the deck of the boat, fish were immediately vented with a trocar and cannula to release gas from the swim bladder and to reduce pressure on internal organs, a wet towel placed over their eyes to protect from direct sunlight, a hose with flowing seawater placed over the gills for irrigation, and opercular respiratory rate constantly monitored during the sampling procedure. We did not see overt clinical reactions or evidence of pain caused by incisions or biopsies. While one fish died prior to release, all others were released immediately after sampling and appeared to swim without evidence of abnormal behavior.

From 2014 to 2017, adult Goliath Grouper were caught off the coasts of Florida, in the Gulf of Mexico and Atlantic Ocean. Fish were sampled during the spawning season from mid-July through September (*n* = 79), which are peak spawning months, but also during the pre-spawning month of May (*n* = 44), where they are preparing to spawn, and in October (*n* = 16) where they are still in spawning condition, but past peak spawning. In four cases, the same fish was caught twice over the 4 years of this study, from independent sampling trips. We treated recaptures of fish as independent samples because it was feasible that sufficient time had passed between captures to allow changes in their physiology.

Tissue and blood samples were collected immediately after fish were transferred to the deck of the boat, including (1) samples of dorsal fin rays (rays 6 and 7 excised at their base) for age determination [methods in (36)]; (2) liver tissue and gill filaments for histopathological analysis; (3) gonad tissue for sex determination [methods in (36)]; and (4) blood for analysis of various health variables.

Dorsal fin rays were immediately placed on ice in the field and stored frozen at −20 °C until they were sent to D. Murie (University of Florida) for age determination <12 months after collection.

Liver tissue (<2 g) was obtained using a sterile stainless-steel biopsy tool inserted through a small incision at the base of the pectoral fin, which was then closed using sterile sutures. For cases where the liver was not easily accessible through the incision, a biopsy was not obtained. A small gill filament biopsy was taken near the edge using sterile surgical scissors. Following collection, tissues were immediately fixed in 10% neutrally-buffered formalin, and, following 24 h of fixation, the samples were rinsed and stored in 70% ethanol and shipped to Crowder Histology (Baton Rouge, Louisiana USA) for processing. Tissues were processed according to standard histological methods using a tissue processor. Processed tissues were embedded in paraffin, sectioned to 3–5 μm thickness, and stained with hematoxylin and eosin (H&E) for viewing by light microscopy. Additional sections were stained with periodic acid Schiff (PAS) with and without diastase.

Gonad biopsies were obtained by inserting a polyethylene catheter into the gonoduct as per previously described techniques (27). Gonads were preserved in formalin and then shipped to D. Murie (University of Florida, Florida USA) for sex determination. Because this is a protogynous hermaphrodite, sexes included males, females, transitionals, or unknown (where insufficient gonad tissue was collected).

At the site of blood collection, scales were removed using a blade and the entire area was repeatedly swabbed with sterile 70% isopropyl alcohol pads. Blood was collected via caudal venipuncture using either a 16- or 18-gauge heparin-coated needle and syringe (Supplemental Figure 1). To heparinize, a liquid solution of heparin sodium was prepared, drawn into each syringe through the needle, and then thoroughly expelled several times prior to blood collection to avoid dilution of blood by heparin. Collected blood was placed into 10 mL lithium-heparin coated BD Vacutainers® (Becton-Dickinson and Co., Franklin Lakes, New Jersey USA) and was carefully inverted to ensure adequate mixture with anticoagulant and subsequently placed over an ice bath in the field for up to 10 h until processing. Hemolysis was minimized by preventing direct contact between blood tubes and the ice. Upon processing blood samples, a portion of whole blood was centrifuged (Fisher Scientific Model 228, Thermo Fisher Scientific, Waltham, Massachusetts USA) at 300 rpm for 10 min to harvest plasma. Plasma aliquots and remaining whole blood were stored frozen at −80°C, with plasma for no more than 12 months, until further analyses were conducted.

### Analysis of Blood Analytes

On the same day as sampling, heparinized whole blood was subsampled in microcapillary tube duplicates and sealed with Critoseal® (Sherwood Medical Co., Deland, Florida USA). Each tube was placed in a microhematocrit centrifuge (Zipocrit® centrifuge, LW Scientific, Lawrenceville, GA, USA) and spun at 11,000 rpm for 5 min. After spinning, packed cell volume (PCV) was assessed as a percentage using a hematocrit microcapillary tube reader. The average of the duplicate samples was reported.

For blood film review, duplicate blood films were prepared for each sample, air-dried, and stained with Wright-Giemsa (Harleco®, EMD Millipore, Billerica, Massachusetts, USA). Blood film evaluation included a white blood cell (WBC) estimate, a 200-WBC differential, and blood cell morphological evaluation.

Frozen plasma samples were sent to the University of Miami Avian and Wildlife Laboratory (UMAW) for protein electrophoresis and quantification of vitellogenin (VTG). Two representative plasma protein electrophoretograms of a female and male Goliath Grouper indicating the 6 fractions of interest are presented in Supplemental Figure 2.

We quantified total protein in two ways: (1) using the Biuret method, indicated as “Total protein (B) in Tables 1–**3**”; and using a refractometric method (ReichertR VET360, Reichert R Technologies, Buffalo, New York USA) indicated as “Total protein (R) in Tables 1–**3**. Electrophoresis was carried out using split beta gels and the SPIFE 3,000 system (Helena Laboratories, Beaumont, Texas USA) to measure the concentrations of 6 fractions.

**Table 1:** Measures of central tendency, range, and reference intervals (with 90% confidence intervals for upper and lower limits) for hematological, plasma biochemical, and plasma protein electrophoretic data for all adult Atlantic Goliath Grouper (*Epinephelus itajara*) in conventional units.

	***N***	**Mean ± SD**	**SE**	**Median**	**Range**	**95% RI (90% CI)**		**Data distribution/RI method/Transformation**
						**Lower limit**	**Upper limit**	
**Hematology**								
Packed cell volume (%)	32	36 ± 7	1	36	24–52	23 (19–26)	50 (46–53)	G/P/N
White blood cells (×10^3^/μL)	140	6.21 ± 1.83	0.15	6.05	2.80–9.50	3.01 (2.80–3.60)	9.30 (9.10–9.50)	NA/NP/N
Neutrophils (×10^3^/μL)	140	1.19 ± 0.53	0.04	1.10	0.29–2.50	0.36 (0.29–0.49)	2.30 (2.10–2.50)	NA/NP/N
Immature neutrophils (×10^3^/μL)	140	0.080.34	0.03	0	0–3.90	0 (0)	0.26 (0.18–3.90)	NA/NP/N
Lymphocytes (×10^3^/μL)	140	3.87 ± 1.14	0.10	3.75	1.60 −6.30	2.05 (1.60–2.20)	5.95 (5.70–6.30)	NA/NP/N
Monocytes (×10^3^/μL)	140	1.08 ± 0.38	0.03	1.00	0.38–2.20	0.47 (0.38–0.53)	1.95 (1.70–2.20)	NA/NP/N
Eosinophils (×10^3^/μL)	140	0.05 ± 0.05	0	0.05	0–0.23	0 (0)	0.18 (0.16–0.23)	NA/NP/N
Basophils (×10^3^/μL)	140	0 ± 0.01	0	0	0–0.08	0 (0)	0 (0–0.08)	NA/NP/N
**Plasma biochemistry**								
Alkaline phosphatase (U/L)	58	68 ± 20	3	65	21–112	28 (21–36)	108 (100–116)	G/P/N
Aspartate aminotransferase (U/L)	58	100 ± 87	11	83	19–652	29 (24–35)	273 (213–353)	G/P/B
Blood urea nitrogen (mg/dL)	58	NA	NA	5	<2–14	<2 (<2)	12 (11–13)	G/P/N
Calcium (mg/dL)	58	15.2 ± 2.9	0.4	14.7	8.3–25.7	8.6 (7.2–10.1)	20.5 (18.9–22.1)	NG/R/N
Calcium: phosphorus ratio	58	1.41 ± 0.34	0.05	1.47	0.77–2.61	0.82 (0.73–0.92)	2.15 (1.99–2.32)	G/P/B
Cholesterol (mg/dL)	58	174 ± 64	8	158	65–432	90 (81–100)	326 (282–378)	G/P/B
Creatine phosphokinase (U/L)	58	NA	NA	148	<20–1,019	<20 (<20–24)	842 (616–1,138)	G/P/B
Creatinine (mg/dL)	58	0.95 ± 0.69	0.09	0.75	0.20–4.10	0.31 (0.27–0.36)	2.62 (2.00–3.50)	G/P/B
Glucose (mg/dL)	58	NA	NA	34	<10–317	NA	NA	NA
Iron (μg/dL)	58	162 ± 50	7	154	56–270	65 (46–83)	259 (241–278)	G/P/N
Lactate dehydrogenase (U/L)	58	2,963 ± 3,619	475	2048	171–25,067	248 (169–365)	13139 (9,008–19,151)	G/P/B
Lipase (U/L)	58	243 ± 141	18	234	11–546	12 (0–40)	561 (490–634)	G/P/B
Magnesium (mg/dL)	58	4.6 ± 2.2	0.3	4.2	2.5–15.6	2.9 (2.8–3.1)	8.8 (7.1–12.3)	G/P/B
Phosphorus (mg/dL)	58	11.3 ± 3.0	0.4	10.9	4.8–18.2	5.5 (4.3–6.6)	17.1 (16.0–18.2)	G/P/N
Potassium (mEq/L)	58	4.8 ± 1.1	0.1	4.8	2.0–7.3	2.6 (2.2–3.0)	7.0 (6.6–7.4)	G/P/N
Sodium (mEq/L)	58	210 ± 43	6	200	131–433	113 (78–160)	290 (243–326)	NG/R/N
Total bilirubin (mg/dL)	58	NA	NA	<0.1	<0.1–0.5	<0.1 (<0.1)	<0.1 (<0.1)	NG/R/N
Triglycerides (mg/dL)	58	96 ± 57	8	85	15–235	18 (12–26)	242 (202–286)	G/P/B
Uric acid (mg/dL)	58	NA	NA	0.5	<0.2–1.8	<0.2 (<0.2)	1.7 (1.4–2.1)	G/P/B
**Plasma proteins**								
Total protein (B) (g/dL)	58	6.0 ± 1.0	0.1	6.0	2.7–8.5	3.9 (3.5–4.3)	8.0 (7.6–8.4)	G/P/N
Total protein (R) (g/dL)	23	5.9 ± 1.0	0.2	5.9	3.7–7.4	4.0 (3.5–4.6)	7.8 (7.2–8.4)	G/P/N
Fraction 1 (g/dL)	58	1.08 ± 0.31	0.04	1.11	0.32–1.98	0.46 (0.34–0.58)	1.69 (1.57–1.81)	G/P/N
Fraction 2 (g/dL)	58	1.22 ± 0.32	0.04	1.17	0.54–1.98	0.58 (0.46–0.71)	1.86 (1.73–1.98)	G/P/N
Fraction 3 (g/dL)	58	0.50 ± 0.16	0.02	0.48	0.21–1.02	0.26 (0.23–0.29)	0.87 (0.77–0.97)	G/P/B
Fraction 4 (g/dL)	58	0.68 ± 0.19	0.03	0.66	0.27–1.34	0.36 (0.31–0.41)	1.11 (1.01–1.21)	G/P/B
Fraction 5 (g/dL)	58	1.91 ± 0.44	0.06	1.88	1.01–3.10	1.04 (0.88–1.21)	2.79 (2.62–2.95)	G/P/N
Fraction 6 (g/dL)	58	0.59 ± 0.23	0.03	0.55	0.16–1.24	0.24 (0.20–0.28)	1.15 (1.01–1.30)	G/P/B

*Mean ± SD was not calculated for analytes where some values fell below the limits of detection; for calculation of reference intervals, values below the limits of detection were assigned to half of the detection limit. Plasma samples with evidence of hemolysis or lipemia were removed from the dataset for calculation of reference intervals. Total protein (R), total protein using refractometer; Total protein (B), total protein by Biuret; G, Gaussian; P, Parametric; NP, Non-parametric; NG, Non-Gaussian; B, Box-Cox; R, Robust; N, None*.

Vitellogenin was quantified using a grouper-specific VTG ELISA kit, which employs the competitive enzyme immunoassay technique in the detection range of 60–1,200 ng/mL (MyBioSource, San Diego, California USA). Vitellogenin was measured by adding standards or samples to appropriate wells containing horseradish peroxidase (HRP) conjugated-rabbit antibody.

Plasma biochemical analytes were measured using the dry chemistry analyzer Vitros 250XR (Ortho, Rochester, New York USA) at UMAW and included alkaline phosphatase (ALP) aspartate aminotransferase (AST), blood urea nitrogen (BUN), calcium, cholesterol, creatine phosphokinase (CK), creatinine, glucose, iron, lactate dehydrogenase (LDH), lipase, magnesium, phosphorus, potassium, sodium, total bilirubin, triglycerides, and uric acid. The calcium:phosphorus ratio was calculated. For glucose, the minimum detection limit was 10 mg/dL. For the two samples that fell below this limit, the low value of “10” was used for statistical purposes.

Immune function, antioxidant capacity, and indicators of oxidative stress were quantified using plasma to measure activities of lysozyme, glutathione peroxidase (GPx), superoxide dismutase (SOD), and concentrations of reactive oxygen and nitrogen species (ROS/RNS). Lysozyme activity was analyzed using standard turbidity assays performed by Walsh et al. (37). At UMAW, GPx was measured using a commercially available glutathione peroxidase assay kit (Cayman Chemical Co., Michigan USA). A spectrophotometer was used to measure GPx activity following the oxidation of NADPH at 340 nm in this reaction. SOD was quantified using a SOD assay kit (Cayman Chemical Co., Ann Arbor, Michigan USA), which detects superoxide radicals generated by xanthine oxidase and hypoxanthine through the use of a tetrazolium salt. SOD was measured as the proportional amount of enzyme needed to exhibit 50% dismutation of the superoxide radical at an absorbance of 450 nm. Concentrations of ROS/RNS were evaluated using an OxiSelectTM *In Vitro* ROS/RNS Assay Kit (Green Fluorescence, Cell Biolabs, Inc., San Diego, California USA), following the manufacturer's instructions (38, 39). This assay universally measured ROS and RON species, which can include, among others, hydrogen peroxide, nitric oxide, peroxynitrite, and peroxyl radicals.

### Histopathological Analysis

Prepared liver and gill tissue slides were evaluated using a Leica DM1000LED microscope (Leica Microsystems Inc, Buffalo Grove, Illinois USA) at UGACVM Histology Laboratory.

Additional analyses on liver tissue slides were conducted at Florida State University (Tallahassee, Florida, USA) to evaluate pigmented macrophage aggregates (PMAs), sometimes referred to as melano-macrophage centers, which are aggregates of highly pigmented phagocytes (40, 41). These were a prominent feature of liver tissue identified from preliminary histological analyses in this study. To quantify area and count of PMAs, images were taken from a subset of the fish with liver biopsies collected (*n* = 55) using a Canon EOS equipped with a DSLR adapter for compound microscopes and Digital Photo Professional 4 software. For each prepared microscope slide, five images were taken of random and non-overlapping representative areas at each 100× and 400× total magnification. Counts of PMAs were quantified manually from 100× images, and area of PMA per total image area in pixels and percent area occupied by PMAs were calculated (42) from 400× images using ImageJ software (43). The five counts and area estimates were then averaged per slide and reported as PMA % area and count (44).

### Data Analysis

#### Reference Intervals

Reference intervals for hematology, plasma biochemistry, and protein electrophoresis data were calculated using MedCalc (MedCalc Software v.18.5, Ostend, Belgium) following the American Society of Veterinary Clinical Pathology reference interval guidelines using a combination of non-parametric and parametric methods, depending on sample size and distribution of the data (45). Reference intervals in conventional units were established for all mature male and female fish combined (Table 1), and also females (Table 2) and males (Table 3) individually. These same reference intervals were also converted to Standard International units (Supplemental Tables 1–3). Normality was assessed using the D'Agostino-Pearson test, while outliers were detected using the Reed test. Logarithmic or Box-Cox transformation was employed when necessary. Biochemistry and protein electrophoresis samples with visual hemolysis scores of ≥1+ were removed from the dataset prior to calculation of reference intervals given the potential interference of hemolysis on various blood analyte measurements (46).

**Table 2:** Measures of central tendency, range, and reference intervals (with 90% confidence intervals for upper and lower limits) for hematological, plasma biochemical, and plasma protein electrophoretic data for mature female Atlantic Goliath Grouper (*Epinephelus itajara*) in conventional units.

	***N***	**Mean ± SD**	**SE**	**Median**	**Range**	**95% RI (90% CI)**		**Data distribution/RI method/Transformation**
						**Lower limit**	**Upper limit**	
**Hematology**								
Packed cell volume (%)	12	38 ± 7	2	39	24–47	NA	NA	Cannot calculate RIs for sample sizes <20
White blood cells (×10^3^/μL)	65	6.07 ± 1.79	0.22	5.90	2.90–9.30	2.39 (1.56–2.89)	9.64 (8.99–10.24)	NG/R/N
Neutrophils (×10^3^/μL)	65	1.16 ± 0.51	0.06	1.10	0.34–2.30	0.39 (0.31–0.48)	2.40 (2.10–2.72)	G/P/B
Immature neutrophils (×10^3^ /μL)	65	0.05 ± 0.09		0	0–0.65	NA	NA	Cannot calculate RIs
Lymphocytes (×10^3^/μL)	65	3.79 ± 1.09	0.13	3.60	2.10–6.00	1.53 (1.23–1.85)	5.98 (5.55–6.33)	NG/R/N
Monocytes (×10^3^/μL)	65	1.07 ± 0.39	0.05	1.00	0.38–2.20	0.31 (0.17–0.45)	1.83 (1.69–1.97)	G/P/N
Eosinophils (×10^3^/μL)	65	0.04 ± 0.05	0.01	0.04	0–0.21	0 (0)	0.13 (0–0.16)	NG/R/N
Basophils (×10^3^/μL)	65	0 ± 0	0	0	0	0 (0)	0 (0)	G/P/N
**Plasma biochemistry**								
Alkaline phosphatase (U/L)	25	63 ± 17	3	58	42–103	29 (19–39)	97 (87–107)	G/P/N
Aspartate aminotransferase (U/L)	25	77 ± 47	9	73	19–262	21 (14–30)	192 (143–255)	G/P/B
Blood urea nitrogen (mg/dL)	25	NA	NA	5	<1–14	<1 (<1–1.7)	12.7 (10.0–15.7)	G/P/B
Calcium (mg/dL)	25	16.6 ± 3.4	0.7	15.7	12.4–25.7	12.3 (11.6–13.1)	26.7 (21.6–38.2)	G/P/B
Calcium:phosphorus ratio	25	1.55 ± 0.36	0.07	1.52	0.77–2.61	0.86 (0.65–1.06)	2.25 (2.04–2.45)	G/P
Cholesterol (mg/dL)	25	192 ± 84	17	167	110–432	99 (88–112)	479 (313–973)	G/P/B
Creatine phosphokinase (U/L)	25	251 ± 257	51	169	22–1019	21 (12–37)	1208 (653–2251)	G/P/B
Creatinine (mg/dL)	25	0.94 ± 0.84	0.17	0.70	0.20–4.10	0.25 (0.19–0.33)	3.07 (1.87–5.43)	G/P/B
Glucose (mg/dL)	25	NA	NA	39	<10–317	NA	NA	NA
Iron (μg/dL)	25	169 ± 54	11	171	87–270	64 (33–95)	275 (244–306)	G/P/N
Lactate dehydrogenase (U/L)	25	3,652 ± 4,964	993	1912	427–25067	293 (182–486)	22096 (9,854–54,385)	G/P/B
Lipase (U/L)	25	169 ± 115	23	185	11–446	3 (0–26)	462 (354–583)	G/P/B
Magnesium (mg/dL)	25	5.3 ± 3.1	0.6	4.0	2.9–15.6	1.6 (1.2–2.5)	10.5 (6.8–13.9)	NG/R/Logarithmic
Phosphorus (mg/dL)	25	11.2 ± 2.9	0.6	10.9	5.6–17.0	5.6 (3.9–7.2)	16.8 (15.1–18.4)	G/P/N
Potassium (mEq/L)	25	5.0 ± 1.0	0.2	5.1	2.7–7.3	3.1 (2.6–3.7)	6.9 (6.3–7.5)	G/P/N
Sodium (mEq/L)	25	224 ± 58	12	209	181–433	82 (25–166)	329 (243–388)	NG/R/N
Total bilirubin (mg/dL)	25	NA	NA	<0.1	<0.1–0.5^a^	<0.1 (<0.1)	<0.1 (<0.1)	NG/R/N
Triglycerides (mg/dL)	25	119 ± 70	14	113	15–235	12 (1.7–31)	290 (228–359)	G/P/B
Uric acid (mg/dL)	25	NA	NA	0.4	<0.2–1.8	<0.2 (<0.2)	1.5 (1.1–2.1)	G/P/B
**Plasma proteins**								
Total protein (B) (g/dL)	25	6.1 ± 1.0	0.2	5.9	4.5–8.5	4.1 (3.5–4.7)	8.0 (7.4–8.6)	G/P/N
Total protein (R) (g/dL)	11	6.0 ± 0.7	0.2	5.9	4.9–7.2	NA	NA	Cannot calculate RIs for sample sizes <20
Fraction 1 (g/dL)	25	1.15 ± 0.33	0.07	1.16	0.32–1.98	0.50 (0.31–0.69)	1.80 (1.61–1.99)	G/P/N
Fraction 2 (g/dL)	25	1.10 ± 0.25	0.05	1.09	0.73–1.59	0.62 (0.47–0.76)	1.59 (1.45–1.74)	G/P/N
Fraction 3 (g/dL)	25	0.56 ± 0.16	0.03	0.54	0.36–1.02^b^	0.28 (0.20–0.36)	0.80 (0.72–0.88)	G/P/N
Fraction 4 (g/dL)	25	0.68 ± 0.21	0.04	0.65	0.41–1.34	0.41 (0.37–0.46)	1.23 (0.97–1.65)	G/P/B
Fraction 5 (g/dL)	25	1.94 ± 0.47	0.09	1.92	1.15–3.10	1.01 (0.74–1.28)	2.86 (2.89–3.14)	G/P/N
Fraction 6 (g/dL)	25	0.64 ± 0.27	0.05	0.60	0.16–1.24	0.10 (0–0.26)	1.18 (1.02–1.34)	G/P/N

a *0.5 mg/dL is an outlier. Reference intervals calculated with this value removed. The next highest value was 0.2 mg/dL*.

b *1.02 g/dL is an outlier. Reference intervals calculated with this value removed. The next highest value was 0.77 g/dL*.

*Mean ± SD was not calculated for analytes where some values fell below the limits of detection; for calculation of reference intervals, values below the limits of detection were assigned to half of the detection limit. Plasma samples with evidence of hemolysis or lipemia were removed from the dataset for calculation of reference intervals. Total protein (R), total protein using refractometer; Total protein (B), total protein by Biuret; G, Gaussian; P, Parametric; NP, Non-parametric; NG, Non-Gaussian; B, Box-Cox; R, Robust; N, None*.

**Table 3:** Measures of central tendency, range, and reference intervals (with 90% confidence intervals for upper and lower limits) for hematological, plasma biochemical, and plasma protein electrophoretic data for mature male Atlantic Goliath Grouper (*Epinephelus itajara*) in conventional units.

	***N***	**Mean ± SD**	**SE**	**Median**	**Range**	**95% RI (90% CI)**		**Data distribution/RI method/Transformation**
						**Lower Limit**	**Upper Limit**	
**Hematology**								
Packed cell volume (%)	20	35 ± 7	2	34	27–52	22 (18–27)	49 (43–54)	G/P/N
White blood cells (×10^3^/μL)	75	6.34 ± 1.86	0.21	6.20	2.80–9.50	3.24 (2.92–3.60)	11.27 (10.15–12.52)	G/P/Logarithmic
Neutrophils (×10^3^/μL)	75	1.22 ± 0.55	0.06	1.10	0.29–2.50	0.39 (0.32–0.48)	2.55 (2.26–2.88)	G/P/B
Immature neutrophils (×10^3^/μL)	75	0.11 ± 0.45	0.05	0	0–3.90	NA	NA	Cannot calculate RIs
Lymphocytes (×10^3^/μL)	75	3.94 ± 1.18	0.14	4.00	1.60–6.30	1.98 (1.78–2.20)	7.11 (6.39–7.92)	G/P/Logarithmic
Monocytes (×10^3^/μL)	75	1.09 ± 0.38	0.04	1.10	0.47–2.00	0.34 (0.22–0.47)	1.83 (1.71–1.96)	G/P/N
Eosinophils (×10^3^/μL)	75	0.06 ± 0.05	0.01	0.06	0–0.23	0 (0)	0.15 (0.13–0.17)	NG/R/N
Basophils (×10^3^/μL)	75	0 ± 0.01	0	0	0–0.08	0 (0)	0 (0)	NG/R/N
**Plasma biochemistry**								
Alkaline phosphatase (U/L)	33	72 ± 22	4	71	21–112	30 (19–40)	115 (104–125)	G/P/N
Aspartate aminotransferase (U/L)	33	117 ± 105	18	90	34–652	42 (36–50)	331 (219–559)	G/P/B
Blood urea nitrogen (mg/dL)	33	7 ± 3	1	6	3–14	3 (2–4)	14 (11–17)	G/P/Logarithmic
Calcium (mg/dL)	33	14.1 ± 1.9	0.3	14.1	8.3–18.6	10.3 (9.3–11.2)	17.9 (16.9–18.9)	G/P/N
Calcium: phosphorus ratio	33	1.31 ± 0.29	0.05	1.39	0.84–2.01	0.74 (0.59–0.88)	1.88 (1.74–2.03)	G/P/N
Cholesterol (mg/dL)	33	161 ± 40	7	153	65–253	83 (64–103)	239 (219–259)	G/P/N
Creatine phosphokinase (U/L)	33	NA	NA	147	<20–728	<20 (<20–25)	643 (454–886)	G/P/B
Creatinine (mg/dL)	33	0.95 ± 0.56	0.10	0.80	0.40–3.30	0.40 (0.35–0.47)	2.26 (1.74–4.21)	G/P/B
Glucose (mg/dL)	33	NA	NA	33	<10–110	NA	NA	NA
Iron (μg/dL)	33	157 ± 46	8	151	56–259	66 (43–89)	248 (224–271)	G/P/N
Lactate dehydrogenase (U/L)	33	2,440 ± 2,049	357	2,081	171–8,577	199 (94–384)	8,607 (6,021–12,032)	G/P/N
Lipase (U/L)	33	299 ± 133	23	304	56–546	38 (0–105)	560 (493–627)	G/P/N
Magnesium (mg/dL)	33	4.2 ± 0.8	0.1	4.2	2.5–6.8	2.8 (2.5–3.1)	5.9 (5.4–6.5)	G/P/B
Phosphorus (mg/dL)	33	11.3 ± 3.1	0.5	10.9	4.8–18.2	5.3 (3.7–6.8)	17.4 (15.9–19.0)	G/P/N
Potassium (mEq/L)	33	4.6 ± 1.2	0.2	4.7	2.0–6.8	2.2 (1.6–2.9)	7.0 (6.4–7.6)	G/P/N
Sodium (mEq/L)	33	200 ± 23	4	198	131–258	156 (144–167)	244 (233–255)	G/P/N
Total bilirubin (mg/dL)	33	NA	NA	<0.1	<0.1–0.3	<0.1 (<0.1)	<0.1 (<0.1–0.2)	NG/R/N
Triglycerides (mg/dL)	33	78 ± 36	6	76	19–153	7 (0–25)	149 (131–167)	G/P/N
Uric acid (mg/dL)	33	NA	NA	0.5	<0.2–1.6	<0.2 (<0.2)	1.5 (1.3–1.7)	G/P/N
**Plasma proteins**								
Total protein (B) (g/dL)	33	5.9 ± 1.1	0.2	6.0	2.7–8.0	3.8 (3.2–4.3)	8.0 (7.5–8.6)	G/P/N
Total protein (R) (g/dL)	12	5.8 ± 1.1	0.3	5.7	3.7–7.4	NA	NA	Cannot calculate RIs for sample sizes <20
Fraction 1 (g/dL)	33	1.02 ± 0.29	0.05	1.08	0.37–1.56	0.45 (0.30–0.59)	1.60 (1.45–1.74)	G/P/N
Fraction 2 (g/dL)	33	1.31 ± 0.35	0.06	1.26	0.54–1.98	0.62 (0.45–0.80)	1.99 (1.82–2.17)	G/P/N
Fraction 3 (g/dL)	33	0.45 ± 0.14	0.02	0.44	0.21–0.87	0.24 (0.20–0.28)	0.77 (0.67–0.89)	G/P/B
Fraction 4 (g/dL)	33	0.69 ± 0.18	0.03	0.67	0.27–1.01	0.33 (0.24–0.42)	1.05 (0.95–1.14)	G/P/N
Fraction 5 (g/dL)	33	1.90 ± 0.43	0.07	1.88	1.01–3.06	1.06 (0.84–1.27)	2.74 (2.52–2.95)	G/P/N
Fraction 6 (g/dL)	33	0.55 ± 0.20	0.03	0.54	0.27–1.08	0.28 (0.24–0.33)	1.05 (0.87–1.29)	G/P/B

*Mean ± SD was not calculated for analytes where some values fell below the limits of detection; for calculation of reference intervals, values below the limits of detection were assigned to half of the detection limit. Plasma samples with evidence of hemolysis or lipemia were removed from the dataset for calculation of reference intervals. Total protein (R), total protein using refractometer; Total protein (B), total protein by Biuret; G, Gaussian; P, Parametric; NP, Non-parametric; NG, NG; B, Box-Cox; R, Robust, N, None*.

#### Statistical Analysis

All analyses were conducted using R (R Development Core Team). To reduce dataset complexity for hematology, plasma biochemistry, and plasma proteins, similar variables were grouped into these respective categories. We used canonical correlation analysis (CCA) as an exploratory analytical method to investigate the relationships between multivariate datasets of size (i.e., total length), age, and sex with health variables in all fish, including juvenile and mature fish and all sexes, to investigate potential morphometrical effects on blood analyte data. Multivariate CCA was carried out separately on analytes of hematology, plasma proteins, and plasma biochemistry to limit the inflation of Type I error rates and to reduce the number of pairwise analyses. Individual parameters of PCV and VTG, along with immune system and oxidative stress, were not tested using CCA due to few parameters relative to the three previously mentioned categories, and lower sample sizes. Tests of dimensionality, to determine which canonical dimensions were statistically significant at the 0.05 level, were conducted using likelihood ratio tests with Wilks' lambda distribution. Zscores were computed to standardize data prior to CCA analyses. Canonical loading values of ~0.3 or greater were used to select the variables determined by this approach to have the most meaningful relationship with the canonical variate (47–49). In R, analyses were carried out using packages CanCorr (http://www.statpower.net/R312/CanCorr.r), CCA, and VEGAN.

To test for the relationships between blood analytes, total length and age, and histological parameters, our analyses were generally restricted to the important variables derived from CCA where CCA was performed. Linear regression analyses were carried out on each clinical and histological variable, including those where CCA was not performed, separately. The only exception to this was for PMA count data with age and size, for which a GLM with family Poisson was conducted. Data were transformed (natural-log, square-root, log-10, cube-root) to meet parametric assumptions where appropriate. Where assumptions were not met for linear regression analysis, packages COIN and lmPerm were used for permutation-based statistical tests with Monte-Carlo simulation as an alternative to classical procedures. The categorical factor “sex” (male, female, transitional) was compared with separate clinical parameters using GLM with family Gaussian. The only exception to this was for PMA count data, for which GLM with family Poisson was conducted. For these GLMs, multiple comparisons were conducted using the glht function with packages emmeans and multcomp to produce Tukey contrasts with adjusted *p*-values. For linear regressions, the Holm-Bonferroni method was used to correct for multiple comparisons (50).

To test the relationship between VTG and each of the six protein fractions determined by protein electrophoretograms, we used linear regression analysis. Data were transformed (natural-log, square-root) to meet parametric assumptions where appropriate.

The relationship between total length (TL, cm) and age (yrs) for Goliath Grouper was previously shown by Malinowski (31) to be strongly positively correlated (*r*^2^ = 0.51, *p* < 2 × 10^−16^), but here we also compared size and age between males, females, and transitionals using analysis of variance and Tukey contrasts with adjusted *p*-values. Because length and age are known to be important factors in determining fish health (51, 52), both were included as factors in this study.

To evaluate trends in blood analytes across months of sample collection for mature males and females, we used analysis of variance and Tukey contrasts with adjusted *p*-values after transforming (natural-log, square-root) any data that did need meet parametric assumptions. For data that did not meet assumptions after transformation, we used GLMs with the glht function for multiple comparisons. Because some months for mature males or females had little data, not all months of sample collection (May, July, August, September, October) were represented for both sexes. For males, there were only two samples from mid-July so we combined those into the month of Aug. This was justifiable because July and August are the two primary spawning months. For females, there was only one sample in October, and we decided to remove from this analysis because it could not be justifiably combined into the previous month.

## Results

### Study Animals and Sampling

A total of 510 Goliath Grouper were caught, from which 195 blood samples were collected for hematological analysis. Of those 195 blood samples, 139 were used for blood plasma analyses of various health variables. Of the 139 samples used for blood plasma analyses, the major portion of this study, they were collected from different sexes and age groups (*n*_*female*_ = 58, *n*_*male*_ = 62, *n*_*transitional*_ = 6, *n*_*unknown*_ = 8, *n*_*juvenile*_ = 5), with ages (yrs) ranging from 4 to 19 (median = 11, mean = 10.8, SD = 3.1) and total lengths (cm) from 57 to 219 (mean = 163.4, median = 165, SD = 27.5) (Supplemental Figure 3). From the 510 fish caught, we also opportunistically collected liver samples (*n* = 183) and gill samples (*n* = 44) for histopathological review, many of which had corresponding blood samples collected, but not all. The number of blood and liver samples collected varied due to logistics of sample collection and handling. A comparison of age and size between sexes for adults showed no difference in age, but a marginally significant difference in total length between males (mean = 163.8, median = 164, SD = 19.2) and females (mean = 173.0, median = 171, SD = 21.2) was observed, with females being larger (*p* = 0.03).

### Reference Intervals

Conventional unit measures of central tendency, range, and reference intervals for all fishes, females only, and males only are reported in Tables 1–3, respectively. Standard International are reported in Tables S1–S3. For plasma biochemical, immune system and oxidative stress analytes with too low of a sample size for reference intervals, descriptive statistics are reported in Table 4.

**Table 4:** Descriptive statistics of immune and oxidative stress indicators, and vitellogenin, for adult (male, female, transitional) Atlantic Goliath Grouper (*Epinephelus itajara*) that were not included as reference intervals.

**Health index**	**Mean**	**SD**	**SE**	**Median**	**Min**	**Max**	**N**
**ALL ADULT**							
**Immune/oxidative stress**							
GPx (nmol/min/ml)	383.25	193.23	28.49	374.39	40.75	784.45	46
Lysozyme (μg/ml)	3.86	1.64	0.19	3.17	1.37	7.74	76
ROS/RNS (nM)	6432.65	1561.47	218.65	6177.36	3431.45	9087.93	51
Superoxide dismutase (U/ml)	15.47	20.16	1.98	7.55	0.82	120.01	104
**Protein precursor**							
Vitellogenin (ng/ml)	433.89	212.33	32.76	382.76	79.32	1017.28	42
**FEMALE**							
**Immune/oxidative stress**							
GPx (nmol/min/ml)	351.98	190.99	42.71	336.19	40.75	652.01	20
Lysozyme (μg/ml)	3.94	1.74	0.30	3.42	1.37	7.11	33
ROS/RNS (nM)	5962.35	1295.73	254.11	5703.19	3431.45	8402.36	26
Superoxide dismutase (U/ml)	15.37	24.06	3.51	5.70	1.33	120.01	47
**Protein precursor**							
Vitellogenin (ng/ml)	506.44	260.21	59.70	469.80	79.32	1017.28	19
**MALE**							
**Immune/oxidative stress**							
GPx (nmol/min/ml)	388.15	207.44	46.38	427.88	40.75	784.45	20
Lysozyme (μg/ml)	3.84	1.60	0.25	3.14	1.78	7.74	41
ROS/RNS (nM)	7010.24	1661.69	339.19	7474.49	3569.95	9087.93	24
Superoxide dismutase (U/ml)	16.95	16.88	2.36	10.32	0.82	79.21	51
**Protein precursor**							
Vitellogenin (ng/ml)	376.82	136.97	33.22	374.35	201.89	784.88	17

*Plasma samples with evidence of hemolysis or lipemia were removed from the dataset for calculation of descriptive statistics. SD, standard deviation; SE, standard error; GPx, glutathione peroxidase; ROS, reactive oxygen species; RNS, reactive nitrogen species*.

### PCV, Hematology, Plasma Proteins, and VTG

There were no significant correlations between hematology data, including PCV and leukogram, and age or total length (*p* > 0.05). Thrombocyte numbers were determined to be adequate in all blood films evaluated, often with variably sized clumps that prevented a more detailed quantitative assessment. Samples from the majority of fish did not show any evidence of red blood cell (RBC) immaturity (i.e., lack of anisocytosis, polychromasia, immature erythroid stages), with only *n* = 3 displaying mild anisocytosis and polychromasia averaging 14 immature RBC/100 mature RBC (range 9–21 immature RBC/100 mature RBC). There were also no significant differences in leukogram data among females, males, and transitionals (*p* > 0.05). Lymphocytes were the predominant WBC type. As described in Materials and Methods, CCA was not performed on PCV or VTG due to few parameters and low sample size.

For plasma proteins, the first CCA (CCA1) was statistically significant (*F* = 2.257, *p* = 0.002) and strongly correlated with a canonical correlation coefficient (CCC) of 0.63 between plasma protein analytes and sex, age, and total length variables (Supplemental Table 5). The total variance explained by the CCA1 was 71.8%. In order of CCA loading value, for plasma protein analytes, the first canonical dimension was most strongly influenced by fraction 2 (loading strength = 0.51), fraction 1 (loading strength = −0.49), and fraction 4 (loading strength = −0.27). For sex, age, and length variables, the first dimension was comprised of total length (loading strength = −0.88) and age (loading strength = −0.46).

Linear regression analysis revealed a number of significant results with protein fractions, which are detailed in Table 5. Fraction 2 was significantly negatively correlated with total length (*r*^2^ = 0.18) and age (*r*^2^ = 0.06), while fraction 4 was negatively correlated with age (*r*^2^ = 0.05). Although sex had a fairly low canonical loading (loading strength = 0.11), we did find that males had significantly higher fraction 2 concentrations than females and that the concentration of this fraction for females was significantly negatively correlated with total length, with this female-only negative correlation (*r*^2^ = 0.25) being stronger than with all sexes combined. The other sex-related result for proteins was that fraction five concentrations were significantly lower in transitionals than both males and females. Concentrations of VTG were similar for males, females, and transitionals (*p* > 0.05), but concentrations significantly increased (*p* < 0.05) with total length (*r*^2^ = 0.26) for females and total length (*r*^2^ = 0.23) for males. PCV did not correlate significantly with total length or age and was not different between sexes (*p* > 0.05).

**Table 5:** Statistically significant plasma protein electrophoresis fractions and vitellogenin of Atlantic Goliath Grouper (*Epinephelus itajara*) categorized by sex (T, transitional; F, female; M, male) and by continuous variables of total length and age.

**Analyte**	Sex (T*F)	(Sex M*F)	Sex (M*T)	**Total length (cm)**	**Age (yr)**
**Plasma protein electrophoresis**					
Fraction 2^a^		*p = 0.005* *n_M/F_ = 37/39* *Z = 3.07*		**p < 0.001** **n = 88, r**^**2**^ **= 0.18** **F = 19.42, df = 86**	**p = 0.009** **n = 86, r**^**2**^ **= 0.06** **F = 6.78, df = 84**
Female				**p < 0.001** **n = 39, r**^**2**^ **= 0.25** **F = 13.31, df = 37**	
Fraction 5	**p = 0.049** **n**_**T/F**_ **= 4/39** **Z = −2.31**		*p = 0.045* *n_M/T_ = 37/4* *Z = 2.35*		
Fraction 4					**p = 0.025** **n = 86, r**^**2**^ **= 0.05** **F = 5.30, df = 84**
**Protein precursor: Vitellogenin**					
Male				**p = 0.030** **n = 18, r**^**2**^ **= 0.23** **F = 6.04, df = 16**	
Female				*p = 0.021* *n = 19, r^2^ = 0.26* *F = 7.43, df = 17*	

a *Data were natural-log transformed*.

*Where male and female categorical variables were significantly different, respective significant post-hoc linear regression results are shown. The p-value is given with adjusted r^2^ value in parentheses. Blank cells indicate no significant difference or correlation. For sex, bolded values indicate the first sex in the pairwise comparison is lower and italicized values indicate the first sex is greater. For total length and age, negative relationships are bolded and positive relationships are italicized*.

There were no significant correlations (*p* > 0.05) between VTG and each of the protein fractions.

**Figure 1:**
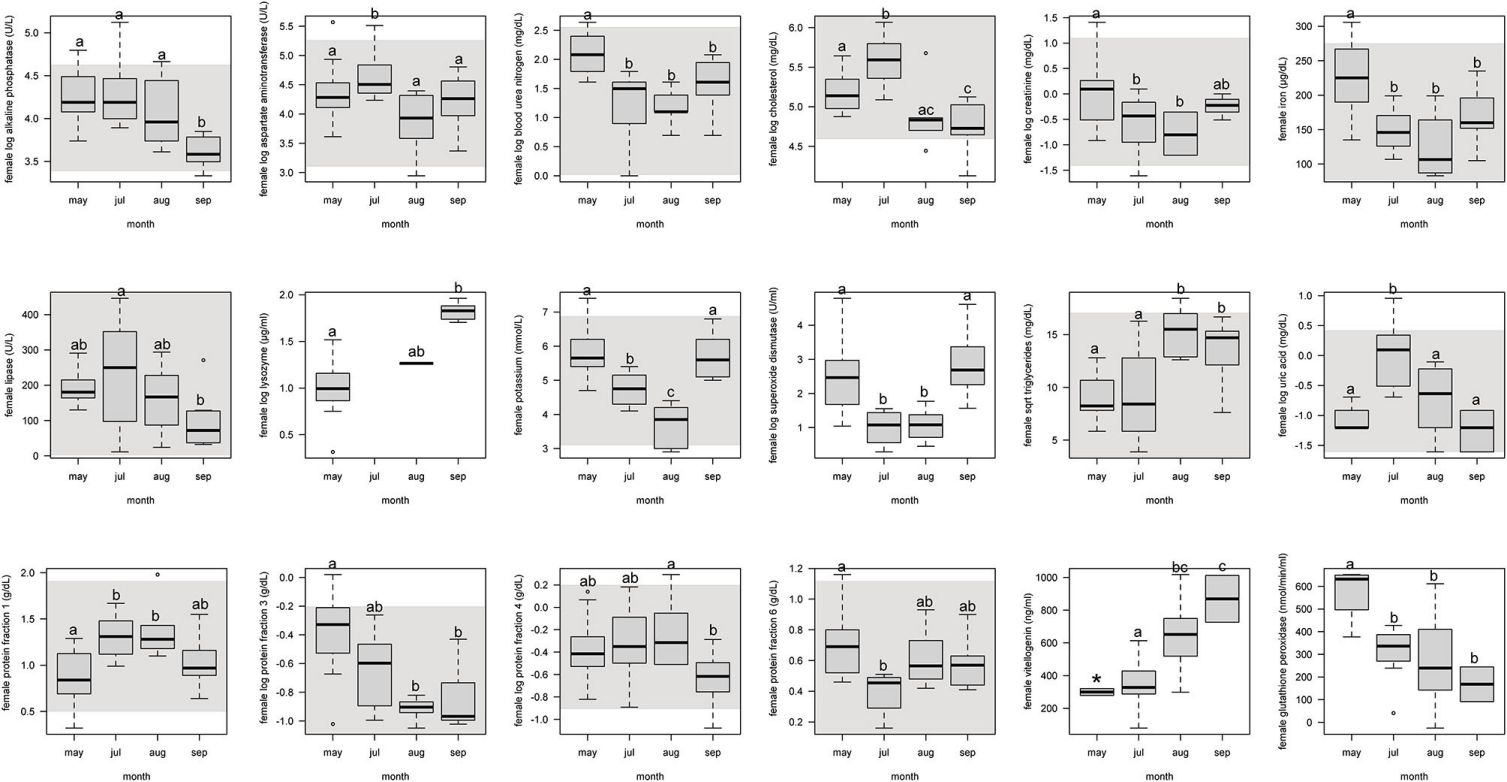
Boxplots showing significant (*p* <0.05) biochemistry analytes, plasma protein electrophoresis fractions, vitellogenin, and immune system and oxidative stress analytes for female Atlantic Goliath Grouper (*Epinephelus itajara*) across sample collection months (May, July, August, September). There was only one sample in October, and we decided to remove from this analysis because it could not be justifiably combined into the previous month. Shading represents reference intervals. Different letters indicate significant difference. Range = vertical dashed lines, median = bold horizontal lines in box, first quartile = area below the line in the box, third quartile = area above the line in the box. The upper and lower ends of the nominal data range is defined as the respective interquartile distance (IQD) ± 1.5 IQD. Open circles = points that fall outside of this range.

For females, protein fraction 1 significantly (*p* < 0.05) increased from May to July and August, and then decreased in September (Figure 1, Supplemental Table 4). Fractions 3 and 6 significantly decreased from May through the following months. Fraction 4 decreased only from September to August. Vitellogenin increased from May through September, but with May not significant because of too few samples (*n* = 2).

**Figure 2:**
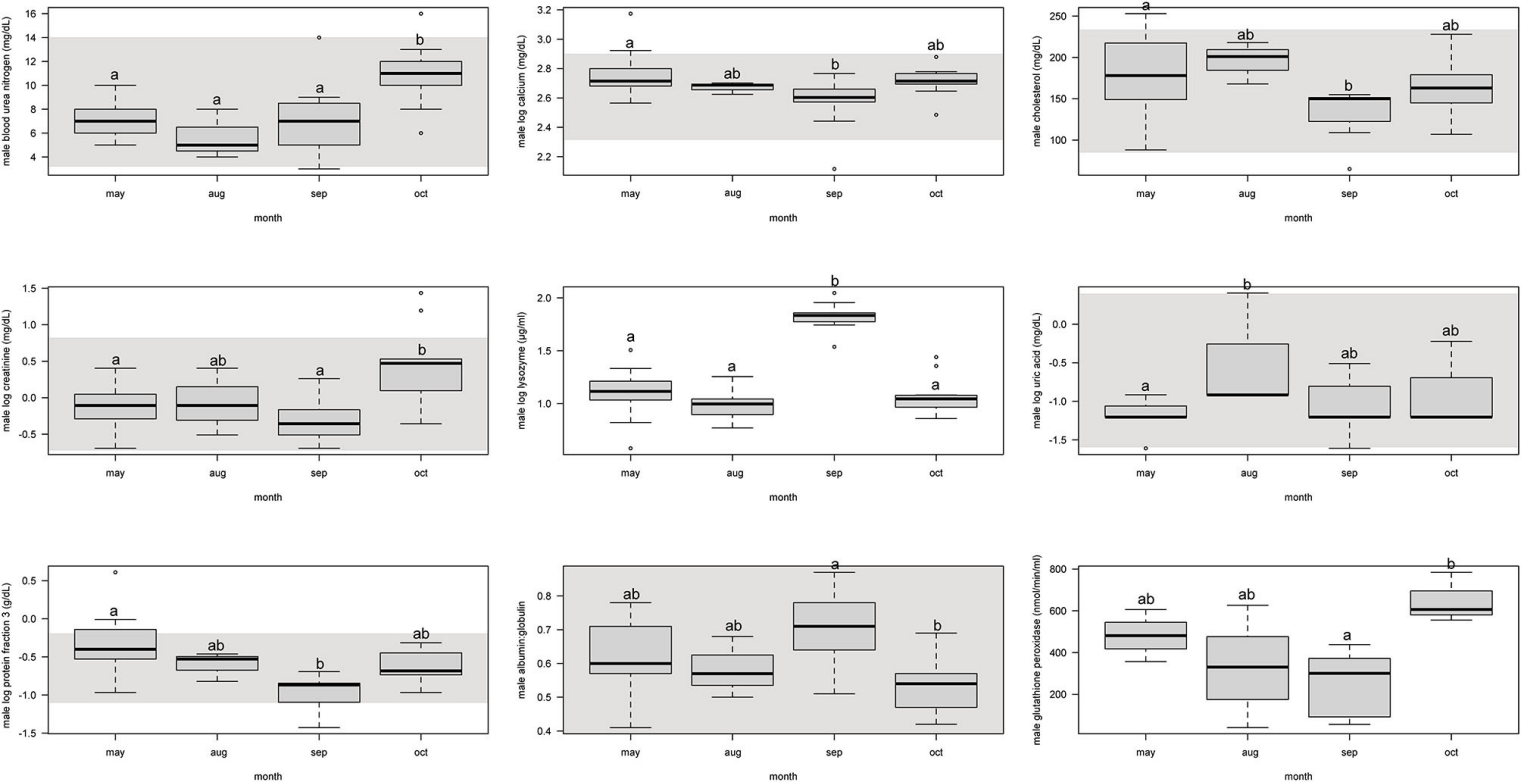
Boxplots showing significant (*p* < 0.05) biochemistry analytes, plasma protein electrophoresis fractions, and immune system and oxidative stress analytes for male Atlantic Goliath Grouper (*Epinephelus itajara*) across sample collection months (May, August, September, October). There were only two samples from mid-July so we combined those into the month of August. Shading represents reference intervals. Different letters indicate significant difference. Range = vertical dashed lines, median = bold horizontal lines in box, first quartile = area below the line in the box, third quartile = area above the line in the box. The upper and lower ends of the nominal data range is defined as the respective interquartile distance (IQD) ± 1.5 IQD. Open circles = points that fall outside of this range.

For males, protein fraction 3 was significantly (*p* < 0.05) lower from May and September, but August and October were not different (Figure 2, Supplemental Table 4).

### Plasma Biochemistry

The CCA for plasma biochemistry included all biochemical analytes. For those, the first canonical correlation was statistically significant (*F*_1_ = 1.72, *p* = 0.007) and strongly correlated with a CCC of 0.76 between blood biochemical analytes and sex, age, and total length variables (Supplemental Table 5). The total variance explained by CCA1 was 56.9%. In order of CCA loading value, for plasma biochemical analytes, the first canonical dimension was most strongly influenced by BUN (loading strength = −0.70), creatinine (loading strength = −0.64), glucose (loading strength = −0.48), uric acid (loading strength = −0.47), lipase (loading strength = −0.45), phosphorus (loading strength = −0.42), LDH (loading strength = −0.36), iron (loading strength = −0.34), CPK (loading strength = −0.30), and potassium (loading strength = −0.26). For sex, age, and total length variables, the first dimension was comprised of total length (loading strength = 0.80), age (loading strength = 0.61), and sex (loading strength = 0.70). Significant results for plasma biochemistry pairwise tests and linear regressions are detailed in Table 6.

**Table 6:** Statistically significant plasma biochemistry analytes of Atlantic Goliath Grouper (*Epinephelus itajara*) categorized by sex (T, transitional; M, male; F, female) and by continuous variables of total length and age.

**Plasma biochemical analyte**	Sex (T*F)	(Sex M*F)	Sex (M*T)	**Total length (cm)**	**Age (yr)**
Blood urea nitrogen		*p = 0.034* *n_M/F_ = 35/38* *Z = 2.46*	*p = 0.002* *n_M/T_ = 35/4* *Z = 3.32*	**p < 0.001** **n = 85, r**^**2**^ **= 0.37** **F = 50.33, df = 83**	**p < 0.001** **n = 83, r**^**2**^ **= 0.22** **F = 23.54, df = 81**
Male				**p < 0.001** **n = 35, r**^**2**^ **= 0.34** **F = 18.56, df = 33**	**p < 0.001** **n = 35, r**^**2**^ **= 0.30** **F = 15.52, df = 33**
Female				**p = 0.04** **n = 38, r**^**2**^ **= 0.08** **F = 4.34, df = 40**	
Calcium	**p = 0.042** **n**_**T/F**_ **= 4/38** **Z = −2.38**				
Creatinine^a^		*p = 0.033* *n_M/F_ = 35/38* *Z = 2.46*	*p = 0.002* *n_M/T_ = 35/4* *Z = 3.32*	**p < 0.001** **n = 85, r**^**2**^ **= 0.32** **F = 39.69, df = 83**	**p = 0.004** **n = 83, r**^**2**^ **= 0.08** **F = 8.05, df = 81**
Male				**p = 0.008** **n = 35, r**^**2**^ **= 0.18** **F = 8.05, df = 32**	**p = 0.031** **n = 35, r**^**2**^ **= 0.11** **F = 5.06, df = 32**
Glucose^a^				**p = 0.004** **n = 85, r**^**2**^ **= 0.08** **F = 7.87, df = 83**	
Iron				**p = 0.013** **n = 84, r**^**2**^ **= 0.08** **F = 8.06, df = 82**	
Lactate dehydrogenase^a^	**p = 0.027** **n**_**T/F**_ **= 4/38** **Z = −2.26**				
Lipase^b^				**p < 0.001** **n = 85, r**^**2**^ **= 0.17** **F = 17.79, df = 83**	**p < 0.001** **n = 83, r**^**2**^ **= 0.10** **F = 10.10, df = 81**
Phosphorus				**p = 0.002** **n = 84, r**^**2**^ **= 0.06** **F = 6.57, df = 82**	**p = 0.004** **n = 83, r**^**2**^ **= 0.06** **F = 6.50, df = 81**
Potassium	**p = 0.003** **n**_**T/F**_ **= 4/38** **Z = −3.28**		*p <0.001* *n_*M*/*T*_ = 35/4* *Z = 3.82*		
Triglycerides^a^		**p = 0.012** **n**_**M/F**_ **= 35/38** **Z = −2.80**			
Uric acid			**p = 0.032** **n**_**M/T**_ **= 35/4** **Z = −2.48**		

a *Data were natural-log transformed*.

b *Data were square-root transformed*.

*Where male and female categorical variables were significantly different, respective significant post-hoc linear regression results are shown. The p-value is given with adjusted r^2^ value in parentheses. Blank cells indicate no significant difference or correlation. For sex, bolded values indicate the first sex in the pairwise comparison is lower and italicized values indicate the first sex is greater. For total length and age, negative relationships are bolded and positive relationships are italicized*.

BUN was significantly (*p* < 0.05) higher in males than females and transitionals. There was a significant (*p* < 0.05) negative relationship between BUN and age for males, but not females, with a stronger correlation for males (*r*^2^ = 0.30) than for all sexes combined (*r*^2^ = 0.22). There was also a significant (*p* < 0.05) negative relationship between BUN and total length for females (*r*^2^ = 0.08) and males (*r*^2^ = 0.34), with males showing the strongest correlation—similar to that of all sexes combined (*r*^2^ = 0.37). Creatinine was significantly (*p* < 0.05) higher in males than females and transitionals, and showed a negative relationship with total length and age. There was a significant (*p* < 0.05) negative relationship between creatinine and age for males, but not females, with a stronger correlation for males (*r*^2^ = 0.11) than for all sexes combined (*r*^2^ = 0.08). There was also a significant (*p* < 0.05) negative relationship between creatinine and total length for males (*r*^2^ = 0.18), with this correlation lower in comparison to all sexes combined (*r*^2^ = 0.32).

Glucose (*r*^2^ = 0.08) and iron (*r*^2^ = 0.0.08) were significantly (*p* < 0.05) negatively correlated with total length, albeit these correlations were weak. Lactate dehydrogenase, potassium, and uric acid all showed significant (*p* < 0.05) differences between sexes. Lactate dehydrogenase activity was significantly lower in transitionals than females, uric acid lower in males than transitionals, and potassium higher in males and females than transitionals (*p* < 0.05 in all cases). Significant (*p* < 0.05) negative relationships occurred for lipase and phosphorus with total length ($r_{\left(lipase\right)}^{2}=0.17$, $r_{\left(phosphorus\right)}^{2}=0.06$) and age ($r_{\left(lipase\right)}^{2}=0.10$, $r_{\left(phosphorus\right)}^{2}=0.06$).

While glucose is an important metabolite that should be considered, we chose to exclude glucose data from reference interval development because samples were negatively affected by sample processing delay, which could not be avoided in this study. The raw data were included so that future investigations could consider confounding effects of sample handling on glucose concentrations by optimizing handling techniques and analytical methodologies.

Calcium (loading strength = −0.15) and triglycerides (loading strength = 0.07) had low CCA loadings (<0.3), but significant (*p* < 0.05) differences between sexes did occur. Calcium was significantly (*p* < 0.05) lower in males than transitionals, while triglycerides were higher in females than males. No other differences or relationships were observed for plasma biochemistry analytes, including ALP, AST, cholesterol, CK, magnesium, sodium, and total bilirubin.

For females, alkaline phosphatase, BUN, creatinine, iron, and lipase significantly (*p* < 0.05) decreased from May through September (Figure 1, Supplemental Table 4). Aspartate aminotransferase, cholesterol, and uric acid significantly increased from the pre-spawning month of May to July, one of the peak spawning months, and then significantly decreased in the following months. Potassium significantly decreased from May through August, but then increased in September. Triglycerides significantly increased from May and July to August and September.

For males, BUN and creatinine significantly (*p* < 0.05) increased from September to October, but were similar to each other in previous months (Figure 2, Supplemental Table 4). Calcium and cholesterol significantly decreased between May and September, but was not different between other months. Uric acid significantly increased from May to August, but the other months were not different.

### Immune System and Oxidative Stress

As described in Materials and Methods, CCA was not performed on factors related to immune system and oxidative stress; however, pairwise tests and linear regressions were conducted and significant results are detailed in Table 7. Reactive oxygen and nitrogen species were significantly (p < 0.05) higher in males than females, and SOD was higher in males than transitionals. Significant (*p* < 0.05) positive linear regressions, albeit fairly weak, occurred for lysozyme with total length (*r*^2^ = 0.13) and age (*r*^2^ = 0.07).

**Table 7:** Statistically significant immune system and oxidative stress analytes of Atlantic Goliath Grouper (*Epinephelus itajara*) categorized by sex (T, transitional; M, male; F, female) and by continuous variables of total length and age.

**Immune/oxidative stress analyte**	Sex (T*F)	(Sex M*F)	Sex (M*T)	**Total length (cm)**	**Age (yr)**
Lysozyme^a^				*p < 0.001* *n = 82, r^2^ = 0.13* *F = 13.41, df = 80*	*p = 0.010* *n = 82, r^2^ = 0.07* *F = 6.93, df = 80*
Superoxide dismutase^a^			*p = 0.049* *n_M/T_ = 51/5* *Z = 2.30*		
Reactive oxygen/nitrogen species		*p = 0.039* *n_M/F_ = 24/26* *Z = 2.38*			

a *Data were natural-log transformed*.

*Where male and female categorical variables were significantly different, respective significant post-hoc linear regression results are shown. The p-value is given with adjusted r^2^ value in parentheses. Blank cells indicate no significant difference or correlation. For sex, bolded values indicate the first sex in the pairwise comparison is lower and italicized values indicate the first sex is greater. For total length and age, negative relationships are bolded and positive relationships are italicized*.

For females, SOD significantly (*p* < 0.05) decreased from May through July and August, and then increased in September (Figure 1, Supplemental Table 4). Glutathione peroxidase significantly decreased from May through September. Lysozyme significantly increased from May through September.

For males, lysozyme significantly increased from August to September, but all other months did not differ from each other (Figure 2, Supplemental Table 4). Glutathione peroxidase significantly increased between September and October, but neither differed from May and August.

### Pigmented Macrophage Aggregates

**Figure 3:**
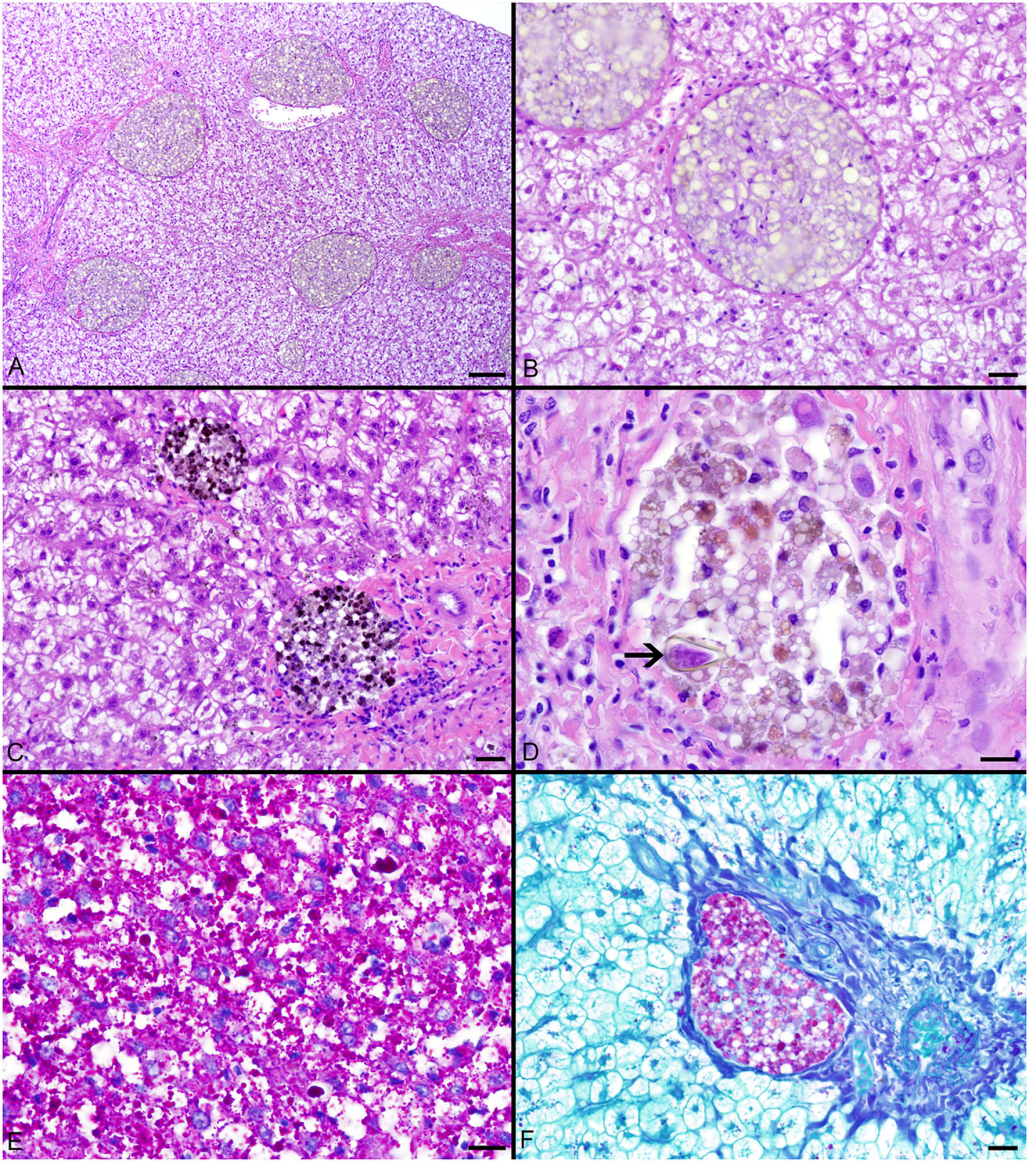
**(A–C)** Hepatocellular vacuolation and variable pigmented macrophage appearance in livers of Atlantic Goliath Grouper (*Epinephelus itajara*). **(D)** High magnification of a pigmented macrophage aggregate shows the presence of a trematode egg (arrow). **(E)** Variably sized globules of intracytoplasmic glycogen within hepatocytes stain deep magenta with PAS. **(F)** Staining of the hepatocellular cytoplasm with PAS is lost after exposure to diastase, consistent with the presence of glycogen, while pigmented macrophage aggregates retain PAS staining, indicating the presence of glycoproteins, particularly lipofuscin. Scale Bars: A = 100 μm, B-C = 20 μm, D-E = 10 μm, F = 20 μm.

Pigmented macrophages formed dense, round to ovoid aggregates with variable yellow, golden brown, and dark brown pigmentation (Figure 3). Total length (range = 57–214 cm) was significantly positively correlated with PMA % area (*r*^2^ = 0.15, *df* = 51, *p* = 0.004) and count (*z* = 2.278, *df* = 51, *p* = 0.02). Age (range = 4–18 yrs) and sex were not significant factors in pigmented macrophage aggregate abundance.

### Other Histological Changes

**Figure 4:**
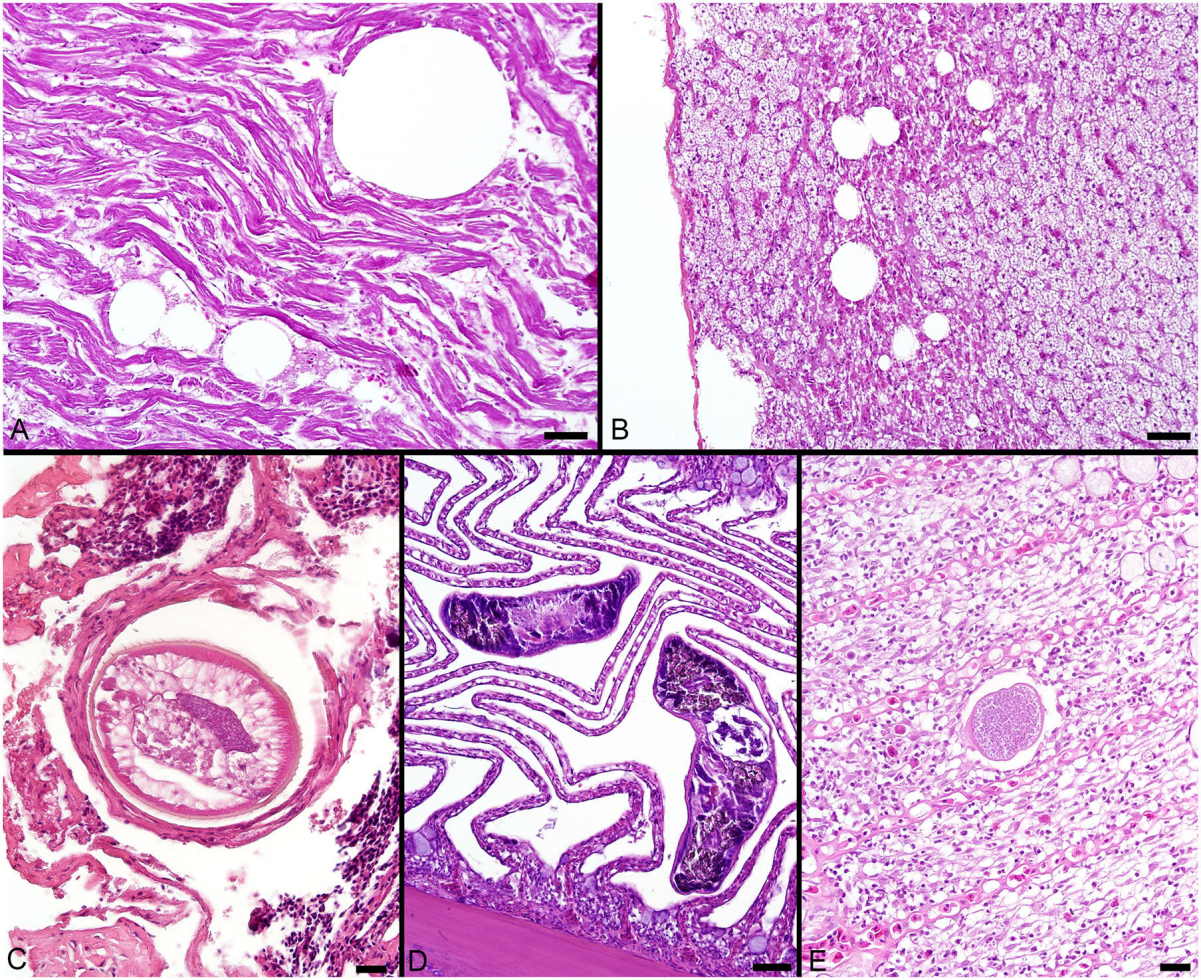
**(A)** Large, round, clear spaces consistent with air bubbles multifocally displace myofibers in the heart of a fish that died during sampling. **(B)** Similar air bubbles obstruct hepatic sinusoids, resulting in congestion of sinusoids and disruption of the adjacent liver parenchyma. **(C)** Cross section through an encysted nematode adjacent to the liver capsule. **(D)** Monogenean parasites found within interlamellar spaces in the gill. **(E)** A single presumptive epitheliocystis inclusion within the gill lamella. Scale Bars: A-B = 50 μm, C = 20 μm, D = 50 μm, E = 20 μm.

The most consistent microscopical change observed was mild to severe, diffuse, indistinct, cytoplasmic vacuolation of hepatocytes, which were PAS positive and diastase sensitive, confirming glycogen content (*n* = 164/183, Figures 3A–F). Rare to infrequent changes (Figures 4A–E) included minimal to mild lymphocytic hepatitis (*n* = 19/183), periductal fibrosis (*n* = 9/183), lipid vacuolation of hepatocytes (*n* = 7/183), mild, mixed periductal and perivascular inflammation (*n* = 4/183), sinusoidal distension by round, clear, well-delineated spaces suggestive of gas bubbles that displace adjacent hepatocytes (*n* = 4/183, Figures 4A,B), and hepatocellular atrophy (*n* = 3/183). In many cases, liver sections contained rare to low numbers of mineralized granulomas (*n* = 72/183). Trematode eggs consisting of a tan, refractile capsule and internal miracidium were rarely to infrequently found within PMAs (*n* = 30/183, Figure 3D). Rare cross sections of encysted nematodes ~120–150 μm in diameter and featuring a ridged, refractile cuticle, coelomyarian musculature, pseudocoelom, lateral cords, intestine, and developing gonads were present within the hepatic parenchyma (*n* = 3/183, Figure 4C). Within the gills, the most frequent changes were rare to low numbers of small, mineralized granulomas (*n* = 11/45), few monogeneans in interlamellar troughs (*n* = 5/45, Figure 4D), and rare, possible epitheliocystis inclusions (*n* = 2/45, Figure 4E).

## Discussion

This study reports novel data on a suite of health variables for Goliath Grouper in Florida waters that provide an essential baseline “snapshot” and an important adjunct tool for assessing population recovery. It also advances our knowledge about Goliath Grouper physiology and lays the groundwork for future research on the effects of various natural and human-induced stressors impinging on this species throughout its range where currently no such baseline health data exists. Given the abundance and novelty of data generated by this work, the focus of this current study lays on the animals and their intrinsic factors as no baseline health data of this kind exist to date on this or other Goliath Grouper populations throughout their range. Future investigations into temporal, spatial, and various other aspects of this population will benefit from the baseline data presented herein.

The influence of intrinsic (e.g., size, age, sex) and extrinsic (e.g., water temperature, habitat, diet, capture techniques) factors on various health variables is well-documented for many species [e.g., (53, 54)]. Intrinsic factors are of particular importance regarding their effects on blood data of fishes (55, 56). Identifying and understanding the influence of these factors, which are more variable and less controllable in wild than managed individuals of the same species, are essential for accurate data interpretation. This is often the biggest challenge in wildlife health assessment studies, given the many logistical challenges in sample acquisition (e.g., stress from capture, effects from anesthesia if applicable) and potential confounding factors related to animal handling and sample collection.

In our study, animal capture and handling techniques, as well as analytical methodologies, were consistent for all analyses, thus minimizing possible variability from extrinsic factors. Therefore, we were able to focus interpretation of health assessment data of Goliath Grouper on morphometrics and time period of sampling, which are among the most important intrinsic and extrinsic factors, respectively. Wildlife health assessments typically include the establishment of reference intervals for blood analytes, and often require partitioning of data into effects from factors such as habitat, season, spawning condition, length, age, and sex when sufficient numbers of samples are available (45, 55, 57). The observed significant difference between males and females in terms of total length, but not age, (Supplemental Figure 3) in this study population needs to be taken into account when considering the discussion of sex differences of various analytes.

While there are a number of blood analyte studies of marine elasmobranchs and cultured marine teleosts [e.g., (10, 15–17, 58)], there are few available for wild populations of marine teleosts (59–61), with the data presented herein being the first for Goliath Grouper off Florida. Hence, it is both challenging and inappropriate to make comparisons among studies that use very different analytical methodologies for species that are vastly different physiologically.

Hematology data for Goliath Grouper showed more consistencies than differences across such intrinsic factors as length, age, and sex. PCV was comparable to other teleost species and fell mostly within reported ranges (62); however, the higher range in Goliath Grouper in this study exceeded the common upper limit of 45% PCV in teleosts, especially in males. This suggests either a subtle sex difference and/or effects from stress during capture and handling (e.g., release of catecholamines resulting in hemoconcentration and swelling of RBCs) (55). Only three fish showed evidence of mild erythroid regeneration, two of which with available PCV data were at the lower end of the reference interval (23 and 24%, respectively). This observation may reflect a recent response to either anemia or transiently lower PCV. Regarding leukogram findings in Goliath Grouper, lymphocytes were the predominant WBC type, which is similar to numerous fish species reported in the literature (55, 62). In addition, very low numbers of immature neutrophils were identified and hence are considered a normal finding in this species.

In contrast to the consistencies with hematology data, plasma biochemical analytes showed several correlations in juvenile and mature fish and monthly trends in mature males and females allowing for considerations regarding some physiological aspects of this species. However, since all correlations of blood analytes with morphometrics were overall weak in this study, these considerations are acceptable if interpreted with caution. Most correlations were observed with length and age concurrently, while few were associated with length alone (e.g., vitellogenin, glucose, iron) and only one with age alone (i.e., protein fraction 4). This shows that this population with an average age of 10.8 yrs (range = 4–19 yrs) and an average total length of 163 cm (range = 57–219 cm) likely represents individuals in phases of somatic growth. Albeit study animals were considered sexually mature, they presumably represent a younger population in the recovery stage, since the oldest Goliath Grouper reported to date was 37 yrs old, and, although the life span of Goliath Grouper remains unknown, this species is thought to live to much older ages (21, 32, 33). Goliath Grouper can also reach sizes of at least 250 cm total length (63), so a majority of individuals in this study population have not yet reached their full growth potential. While this study provides a baseline for morphometrical data in this recovering population of Goliath Grouper, to see if these patterns and relationships hold in older and larger Goliath Grouper that reach their full growth and age potential, continued state and federal protection from lethal extraction of this species is necessary. In addition, analyte differences (e.g., fraction 2 “presumptive albumin,” BUN) in males and females may be associated with effects of sexual maturation or other associated dietary or physiological changes (e.g., tissue growth). Males mature at slightly smaller sizes and younger ages than females (21, 35), and the spawning season, when many samples were collected, is the only time of year where females would transition to males. Moreover, most individuals from this population transition within the mean age range of individuals in this study (25). The many variations of blood analytes in mature males and females across sampled months suggest sex-specific metabolic and physiological differences in pre-spawning and spawning months in this species.

Several identified differences in plasma biochemical analytes with length, age and/or sex indicate potential significance regarding tissue growth and metabolic and/or nutritional changes in a growing, reproductively active population. These analytes include BUN and phosphorus, which were negatively correlated with length and age, possibly indicating a difference in feeding intensity or protein metabolism. However, a recent meal before capture should also be considered. Higher BUN and creatinine in males compared to females and transitionals, but lower uric acid in males compared to transitionals suggest sex differences in muscle mass and/or protein/energy metabolism. This is further supported by variations in BUN, creatinine, and uric acid between adult males and females across sampled months. Although fish primarily excrete nitrogenous waste as ammonia across their gills, urea is still present in all fish, if only comprising a low percentage of nitrogenous waste, and is often of research interest, along with creatinine, in diagnosing gill or liver disease (64, 65). Future Goliath Grouper research should consider these patterns along with environmental toxicants in diagnosing disease or other negative health effects. The negative correlation of creatinine in males with length and age may suggest that smaller males possibly have higher muscle mass or reflect tissue growth. Creatinine, although similar to BUN in that it occurs in relatively small amounts in teleosts, can also be a good indicator of health and potential renal damage (64). The excretion and metabolism of nitrogenous wastes in fishes is still poorly understood and the clinical and physiological significance of these analytes still remains unknown (55). Observed correlations, sex differences, and monthly trends of other analytes including calcium, cholesterol, iron, enzymes, and potassium may suggest subtle differences or variations in feeding frequency/fasting, diet, metabolic rates, reproductive physiology, or nutritional state of individuals prior to sample collection. In this same Goliath Grouper study population, a concurrent study found that males and females consumed the same prey but at different frequencies, and there was a lot of variation in stomach fullness, with many individuals having empty stomachs at time of capture (66). Future research should directly consider diet and blood plasma analytes to better understand the relationship between these two factors.

The protein electrophoretogram of the Goliath Grouper consistently provided 6 fractions across the analyses of all study samples. The fraction migration was very similar to that seen in mammals with a dominant fraction 2 (presumptive albumin) and prominent fraction 5 (presumptive beta globulin migrating fraction). Previously, 6 and 5 fractions have been described in Rainbow Trout *Oncorhynchus mykiss* (67) and Koi *Cyprinus carpio* (68), respectively. Whereas, Koi electrophoretogram also had dominant fraction 2 and 5, Rainbow Trout samples exhibited a dominant fraction 3. Assigning specific protein fraction labels to fraction 1 to 6 is difficult as very few studies have been conducted on teleosts using modern protein electrophoresis methods. Changes in acute phase proteins in fish with natural or experimental infection and with inflammatory processes have been documented (69) so it is likely that many of these fractions represent proteins, metabolites, and hormones that function similarly to their mammalian counterparts. This conclusion is further supported by the observed variations in protein fractions across months in mature males and females. The negative correlation of fraction 2 with length and age is likely due to the observed significantly lower concentrations in females and the negative correlation for females by length. This sex difference corresponds with trends in other analytes, such as higher BUN and creatinine in males, suggesting differences in protein metabolism by sex. Fraction 4 is the one analyte showing a negative correlation only with age. This is an interesting contrast to the positive correlation of lysozyme with length and age, indicating a tendency to increase in growing Goliath Grouper, potentially suggestive of a greater capacity of the innate immune system and/or greater cumulative exposure to antigens as these fish age. Sex differences were observed through differences of higher SOD in males compared to transitionals, higher ROS/RNS species in males compared to females, and monthly trends of lysozyme, SOD, and GPx in mature males and females, possibly suggesting variations in energy balance, diet, or an association with circulating reproductive proteins.

Vitellogenin was positively correlated with length in females and negatively in males, with a substantial increase in mature females from pre-spawning through spawning months, showing an overt sex difference in a maturing population with growth and sexual development. For a protogynous hermaphrodite (i.e., change sex from female to male), like Goliath Grouper, it would also make sense that any males that transitioned from females would initially have high VTG that would then be reduced as the now-male continues to grow, since VTG is an egg yolk precursor protein normally found only in females (70, 71). Individuals for this health assessment study are well within the range of sizes and ages of transitional females observed for this population, which were shown in a previous study to range from 108 to 191 cm TL and 4 to 12 yrs of age (25). However, circulating VTG can change rapidly, as has been shown in other grouper species (72), and so changes in plasma VTG during and after sex change in protogynous species should be a focus of future studies. Similar to the pattern in VTG, triglycerides were higher in females compared to males, suggesting a difference in circulating lipids and lipoproteins needed for vitellogenesis (73, 74). Although there was overlap in plasma VTG concentrations in males and females, these findings suggest possible utility of VTG as a non-invasive biomarker for sex determination in consideration of size of fish and month of sampling given that analytical techniques are available via ELISA and proteomics (72, 75). Since VTG did not correlate with any of the plasma protein fractions, traditional protein electrophoresis is presumably insensitive for the identification of VTG variations in this species. The baseline data presented herein may be useful for future studies of the effects of environmental estrogens and reproductive success (76, 77).

Hepatocyte morphology and appearance in fish liver tissue may vary considerably based on species, age, sex, season, nutritional status, and exposure to environmental pollutants (78). The histological changes described in this study reflect either typical, species-related observations, findings associated with life stage or active reproduction, or subclinical to clinically insignificant infectious and/or inflammatory processes. The observed positive correlation of PMA % area and count with total length shows an increase as these fish grow and mature, presumptively from continuous active inflammation (e.g., antigen exposure) and/or metabolic changes over time. The main finding of prominent vacuolation of hepatocytes was consistent with abundant, intracytoplasmic glycogen storage, which was confirmed by diastase sensitive PAS staining. Excess energy intake beyond basal metabolism and other demands is often stored in fish hepatocytes as glycogen and/or lipid and utilized in fasting situations (79–81). Fish species physiology and diet components may predispose the accumulation of glycogen vs. lipid (79–81). Glycogen accumulation is frequently seen in the hepatocytes of captive and occasionally wild fish, particularly trout and seabass, which may reflect carbohydrate rich diets, minimal energy expenditure for foraging, and/or poor utilization of dietary carbohydrates (78–81). Few fish had vacuolation consistent with lipid type which may suggest either mobilization of lipid from adipose stores (e.g., associated with reproduction), excess dietary lipid, or, much less likely, hepatocellular injury and dysfunction. Given the uniformity and diffuse nature of vacuolation and lack of associated tissue or cellular alterations, the vacuolation likely represents typical, physiological glycogen and/or lipid storage for this species (79, 80). Infectious agents, when present, were in very low numbers and likely had little to no impact on the fish. There was no indication of neoplastic or other proliferative processes in the samples.

Microscopical gas bubbles within hepatic sinusoids were suggestive of peracute gas supersaturation similar to decompression sickness rather than traditional gas bubble disease typically seen in fish exposed to excess dissolved gases in water (82). These changes were considered iatrogenic, and potentially due to capture methods bringing the fish up from depth, removal of surrounding pressure, and release of dissolved gases in the blood and highly vascularized tissues (82). This last result is highly important for consideration of the health of Goliath Grouper in the current catch-and-release fishery that has grown into a major non-consumptive enterprise in Florida (32), in addition to optimizing fish capture techniques for health assessment studies. Although a catch-and-release fishery is a far better alternative to that of an extractive fishery, stress and other patho-/physiological effects need to be further considered as this enterprise continues to expand, particularly related to barotrauma and how their dissolved blood gases shift out of solution, resulting in decompression-like embolisms as the fish is pulled rapidly to the surface. For research purposes, and to better understand these effects while minimizing the stress on the animal, improvement to sampling techniques, as we have done in the past, would involve venting swim bladders mid-water prior to surfacing to minimize barotrauma, and/or using descending devices to safely return fish back to depth where expanded gases can contract [e.g., (83)].

This study is the first to report a suite of various health indices of the Atlantic Goliath Grouper off the coasts of Florida, several of which indicate physiologically-relevant differences in length, age, and sex. It adds to the otherwise sparse literature of hematological, biochemical, immune function, and oxidative stress data in free-ranging populations of marine teleost fishes. It also demonstrates that non-invasive blood sampling provides an opportunity to obtain critical endpoint data and a vast amount of health information in a species in need of population and physiological monitoring. Future studies might include additional diagnostic methodologies (e.g., for parasitology, microbiology) that were beyond the scope of this study. Additionally, our study adds to existing data on high muscle and liver tissue concentrations of mercury in this species that are among the highest of any teleost in the Atlantic or Gulf of Mexico (31). The current study provides the necessary health baselines for which to compare health effects related to tissue contaminant concentrations. The health indices reported herein will be critical for monitoring dynamics and demographics of the Florida population and for understanding physiological responses to various stressors and to a changing global oceanic environment specifically relevant to this population and associated organisms within their ecosystem (1). Further, it will facilitate the identification of conservation problems and challenges pertaining to this species and its habitat and provide the opportunity for targeted conservation efforts by state and federal agencies.

## Data Availability Statement

The raw data supporting the conclusions of this article will be made available by the authors, without undue reservation.

## Ethics Statement

The animal study was reviewed and approved by Florida State University Institutional Animal Care and Use Committee (IACUC) (protocol #s: 1106, 1411, and 1718).

## Author Contributions

CM: study design, setup and organization of sampling protocols, field collections and sample processing, preservation and transport, data analyses, histological and blood plasma sample analyses, manuscript writing and editing, and manuscript submission. JP: setup and organization of sampling protocols, blood plasma sample analyses, manuscript writing and editing, and reference intervals. NS: manuscript writing and editing, data interpretation, and blood film review. JS: histopathological analysis, and manuscript writing and editing. CC: blood plasma sample analyses, and manuscript writing and editing. FC and CK: manuscript writing and editing, sample collection protocol, and general project oversight. All authors contributed to the article and approved the submitted version.

## Conflict of Interest

The authors declare that the research was conducted in the absence of any commercial or financial relationships that could be construed as a potential conflict of interest.
