# Supplementary material for: The Iconic Atlantic Goliath Grouper (Epinephelus itajara): A Comprehensive Assessment of Health Indices in the Southeastern United States Population
Source: Front Vet Sci. 2020 Sep 25;7:635. doi: 10.3389/fvets.2020.00635 (PMC7546827; doi:10.3389/fvets.2020.00635)
Supplement: Supplementary file 2 [file Table_2.docx]

| **Supplemental Table 2.** Measures of central tendency, range, and reference intervals (with 90% confidence intervals for upper and lower limits) for hematological, plasma biochemical, and plasma protein electrophoretic data for mature female Atlantic Goliath Grouper (*Epinephelus itajara*) in Standard International units. Mean±SD was not calculated for analytes where some values fell below the limits of detection; for calculation of reference intervals, values below the limits of detection were assigned to half of the detection limit. Plasma samples with evidence of hemolysis or lipemia were removed from the dataset for calculation of reference intervals. Note: Total protein (R) = total protein using refractometer, Total protein (B) = total protein by Biuret. | | | | | | | | | |
| --- | --- | --- | --- | --- | --- | --- | --- | --- | --- |
|  | | **N** | **Mean±SD** | **SE** | **Median** | **Range** | **95% RI (90% CI)** | | **Data distribution/RI method/Transformation** |
|  |  |  |  |  |  |  | **Lower Limit** | **Upper Limit** |  |
| **Hematology** | | | | | | | | |  |
| Packed cell volume (%) | | 12 | 38±7 | 2 | 39 | 24–47 | NA | NA | Cannot calculate RIs for sample sizes <20 |
| White blood cells (x 10^3^ cells/μL) | | 65 | 6.07±1.79 | 0.22 | 5.90 | 2.90–9.30 | 2.39 (1.56–2.89) | 9.64 (8.99–10.24) | Non-Gaussian/Robust/None |
| Neutrophils (x 10^3^ cells/μL) | | 65 | 1.16±0.51 | 0.06 | 1.10 | 0.34–2.30 | 0.39 (0.31–0.48) | 2.40 (2.10–2.72) | Gaussian/Parametric/Box-Cox |
| Immature neutrophils (x 10^3^ cells/μL) | | 65 | 0.05±0.09 |  | 0 | 0–0.65 | NA | NA | Cannot calculate RIs |
| Lymphocytes (x 10^3^ cells/μL) | | 65 | 3.79±1.09 | 0.13 | 3.60 | 2.10–6.00 | 1.53 (1.23–1.85) | 5.98 (5.55–6.33) | Non-Gaussian/Robust/None |
| Monocytes (x 10^3^ cells/μL) | | 65 | 1.07±0.39 | 0.05 | 1.00 | 0.38–2.20 | 0.31 (0.17–0.45) | 1.83 (1.69–1.97) | Gaussian/Parametric/None |
| Eosinophils (x 10^3^ cells/μL) | | 65 | 0.04±0.05 | 0.01 | 0.04 | 0–0.21 | 0 (0) | 0.13 (0–0.16) | Non-Gaussian/Robust/None |
| Basophils (x 10^3^ cells/μL) | | 65 | 0±0 | 0 | 0 | 0 | 0 (0) | 0 (0) | Gaussian/Parametric/None |
| **Plasma biochemistry** | | | | | | | | | |
| Alkaline phosphatase (U/L) | | 25 | 63±17 | 3 | 58 | 42–103 | 29 (19–39) | 97 (87–107) | Gaussian/Parametric/None |
| Aspartate aminotransferase (U/L) | | 25 | 77±47 | 9 | 73 | 19–262 | 21 (14–30) | 192 (143–255) | Gaussian/Parametric/Box-Cox |
| Blood urea nitrogen (mmol/L) | | 25 | NA | NA | 1.79 | <0.36–5.00 | <0.36 (<0.36–0.61) | 4.53 (3.57–5.61) | Gaussian/Parametric/Box-Cox |
| Calcium (mmol/L) | | 25 | 2.4±0.9 | 0.2 | 3.9 | 3.1–6.4 | 3.1 (2.9–3.3) | 6.7 (5.4–9.6) | Gaussian/Parametric/Box-Cox |
| Calcium:phosphorus | | 25 | 1.20±0.28 | 0.06 | 1.17 | 0.59–2.02 | 0.66 (0.50–0.82) | 1.74 (1.58–1.90) | Gaussian/Parametric/None |
| Cholesterol (mmol/L) | | 25 | 5.0±2.2 | 0.4 | 4.4 | 2.9–11.2 | 2.6 (2.3–2.9) | 12.4 (8.1–25.2) | Gaussian/Parametric/Box-Cox |
| Creatine phosphokinase (U/L) | | 25 | 251±257 | 51 | 169 | 22–1019 | 21 (12–37) | 1208 (653–2251) | Gaussian/Parametric/Box-Cox |
| Creatinine (µmol/L) | | 25 | 83.1±74.3 | 15.0 | 61.9 | 17.7–362.4 | 22.1 (16.8–29.2) | 271.4 (165.3–479.5) | Gaussian/Parametric/Box-Cox |
| Glucose (mmol/L) | | 25 | NA | NA | 2.2 | <0.6–17.6 | <0.6 (<0.6) | 33.5 (8.7–28.8) | Gaussian/Parametric/Box-Cox |
| Iron (μmol/L) | | 25 | 30.3±9.7 | 2.0 | 30.6 | 15.6–48.3 | 11.5 (5.9–17.0) | 49.9 (43.7–54.8) | Gaussian/Parametric/None |
| Lactate dehydrogenase (U/L) | | 25 | 3652±4964 | 993 | 1912 | 427–25067 | 293 (182–486) | 22096 (9854–54385) | Gaussian/Parametric/Box-Cox |
| Lipase (U/L) | | 25 | 169±115 | 23 | 185 | 11–446 | 3 (0–26) | 462 (354–583) | Gaussian/Parametric/Box-Cox |
| Magnesium (mmol/L) | | 25 | 2.2±1.3 | 0.3 | 1.7 | 1.2–6.4 | 0.7 (0.5–1.0) | 4.3 (2.8–5.7) | Non-Gaussian/Robust/Logarithmic |
| Phosphorus (mmol/L) | | 25 | 3.6±0.9 | 0.2 | 3.5 | 1.8–5.5 | 1.8 (1.3–2.3) | 5.4 (4.9–5.9) | Gaussian/Parametric/None |
| Potassium (mmol/L) | | 25 | 5.0±1.0 | 0.2 | 5.1 | 2.7–7.3 | 3.1 (2.6–3.7) | 6.9 (6.3–7.5) | Gaussian/Parametric/None |
| Sodium (mmol/L) | | 25 | 224±58 | 12 | 209 | 181–433 | 82 (25–166) | 329 (243–388) | Non-Gaussian/Robust/None |
| Total bilirubin (µmol/L) | | 25 | NA | NA | <1.7 | <1.7–8.6^a^ | <1.7 (<1.7) | <1.7 (<1.7) | Non-Gaussian/Robust/None |
| Triglycerides (mmol/L) | | 25 | 1.34±0.79 | 0.16 | 1.28 | 0.17–2.66 | 0.14 (0.02–0.35) | 3.28 (2.58–4.06) | Gaussian/Parametric/Box-Cox |
| Uric acid (mmol/L) | | 25 | NA | NA | 0.02 | <0.01–0.11 | <0.01 (<0.01) | 0.09 (0.07–0.12) | Gaussian/Parametric/Box-Cox |
| **Plasma proteins** | | | | | | | | | |
| Total protein (B) (g/L) | | 25 | 61±10 | 2 | 59 | 45–85 | 41 (35–47) | 80 (74–86) | Gaussian/Parametric/None |
| Total protein (R) (g/L) | | 11 | 60±7 | 2 | 59 | 49–72 | NA | NA | Cannot calculate RIs for sample sizes <20 |
| Fraction 1 (g/L) | | 25 | 11.5±3.3 | 0.7 | 11.6 | 3.2–19.8 | 5.0 (3.1–6.9) | 18.0 (16.1–19.9) | Gaussian/Parametric/None |
| Fraction 2 (g/L) | | 25 | 11.0±2.5 | 0.5 | 10.9 | 7.3–15.9 | 6.2 (4.7–7.6) | 15.9 (14.5–17.4) | Gaussian/Parametric/None |
| Fraction 3 (g/L) | | 25 | 5.6±1.6 | 0.3 | 5.4 | 3.6–10.2^b^ | 2.8 (2.0–3.6) | 8.0 (7.2–8.8) | Gaussian/Parametric/None |
| Fraction 4 (g/L) | | 25 | 6.8±2.1 | 0.4 | 6.5 | 4.1–13.4 | 4.1 (3.7–4.6) | 12.3 (9.7–16.5) | Gaussian/Parametric/Box-Cox |
| Fraction 5 (g/L) | | 25 | 19.4±4.7 | 0.9 | 19.2 | 11.5–31.0 | 10.1 (7.4–12.8) | 28.6 (28.9–31.4) | Gaussian/Parametric/None |
| Fraction 6 (g/L) | | 25 | 6.4±2.7 | 0.5 | 6.0 | 1.6–12.4 | 1.0 (0–2.6) | 11.8 (10.2–13.4) | Gaussian/Parametric/None |
| ^a^ 8.6 µmol/L is an outlier. Reference intervals calculated with this value removed. The next highest value was 3.4 µmol/L.  ^b^ 10.2 g/L is an outlier. Reference intervals calculated with this value removed. The next highest value was 7.7 g/L. | | | | | | | | | |
|  |  | | | | | | | |  |
