# Supplementary material for: The Iconic Atlantic Goliath Grouper (Epinephelus itajara): A Comprehensive Assessment of Health Indices in the Southeastern United States Population
Source: Front Vet Sci. 2020 Sep 25;7:635. doi: 10.3389/fvets.2020.00635 (PMC7546827; doi:10.3389/fvets.2020.00635)
Supplement: Supplementary file 5 [file Table_5.docx]

| **Supplemental Table 5**. Significant (p < 0.05) canonical structures of the first pair of canonical variates for (a) plasma proteins; and (b) plasma biochemistry analytes of Atlantic Goliath Grouper (*Epinephelus itajara*). Analytes and total length, age, and sex tests with standardized canonical coefficient loadings >0.3, our cutoff limit, are bold and are presented in descending order. Rc1 = the first canonical correlation. The total variance explained for each CCA 1 axis is presented below. | | | |
| --- | --- | --- | --- |
| *Plasma protein electrophoresis* | | | |
| First pair, *Rc_1_* = 0.63 (Approx. *F* = 2.26; *P* = 0.002) | | | |
| *Analyte* | | *Sex, age, total length* | |
| Variables | Loading | Variables | Loading |
| **Fraction 2** | **0.5086** | **Total length** | –0.8801 |
| **Fraction 1** | **–0.4868** | **Age** | –0.4643 |
| **Fraction 4** | **–0.2695** | Sex | 0.1069 |
| Fraction 5 | 0.1174 |  |  |
| Fraction 6 | –0.0337 |  |  |
| Fraction 3 | –0.0312 |  |  |
| Total Protein | –0.0004 |  |  |
| CCA1 total variance explained: 71.8% | | | |
| *Plasma biochemistry* | | | |
| First pair, *Rc­_1_* = 0.7570 (Approx. *F* = 1.7178; *P* = 0.007 | | | |
| *Analyte* | | *Sex, age, total length* | |
| Variables | Loading | Variables | Loading |
| **Blood urea nitrogen** | **–0.7048** | **Total length** | **0.8040** |
| **Creatinine** | **–0.6422** | **Age** | **0.0611** |
| **Glucose** | **–0.4823** | **Sex** | **0.6971** |
| **Uric acid** | **–0.4749** |  |  |
| **Lipase** | **–0.4512** |  |  |
| **Phosphorus** | **–0.4167** |  |  |
| **Lactate dehydrogenase** | **–0.3609** |  |  |
| **Iron** | **–0.3426** |  |  |
| **Creatine phosphokinase** | **–0.2967** |  |  |
| **Potassium** | **–0.2566** |  |  |
| **Bilirubin** | **–0.2522** |  |  |
| Calcium | –0.1546 |  |  |
| Alkaline phosphatase | –0.1369 |  |  |
| Sodium | –0.1317 |  |  |
| Magnesium | –0.1109 |  |  |
| Triglycerides | 0.0654 |  |  |
| Cholesterol | –0.0249 |  |  |
| Aspartate aminotransferase | –0.0223 |  |  |
| CCA1 total variance explained: 56.9% | | | |
