# Supplementary material for: The Iconic Atlantic Goliath Grouper (Epinephelus itajara): A Comprehensive Assessment of Health Indices in the Southeastern United States Population
Source: Front Vet Sci. 2020 Sep 25;7:635. doi: 10.3389/fvets.2020.00635 (PMC7546827; doi:10.3389/fvets.2020.00635)
Supplement: Supplementary file 6 [file Image_1.pdf]

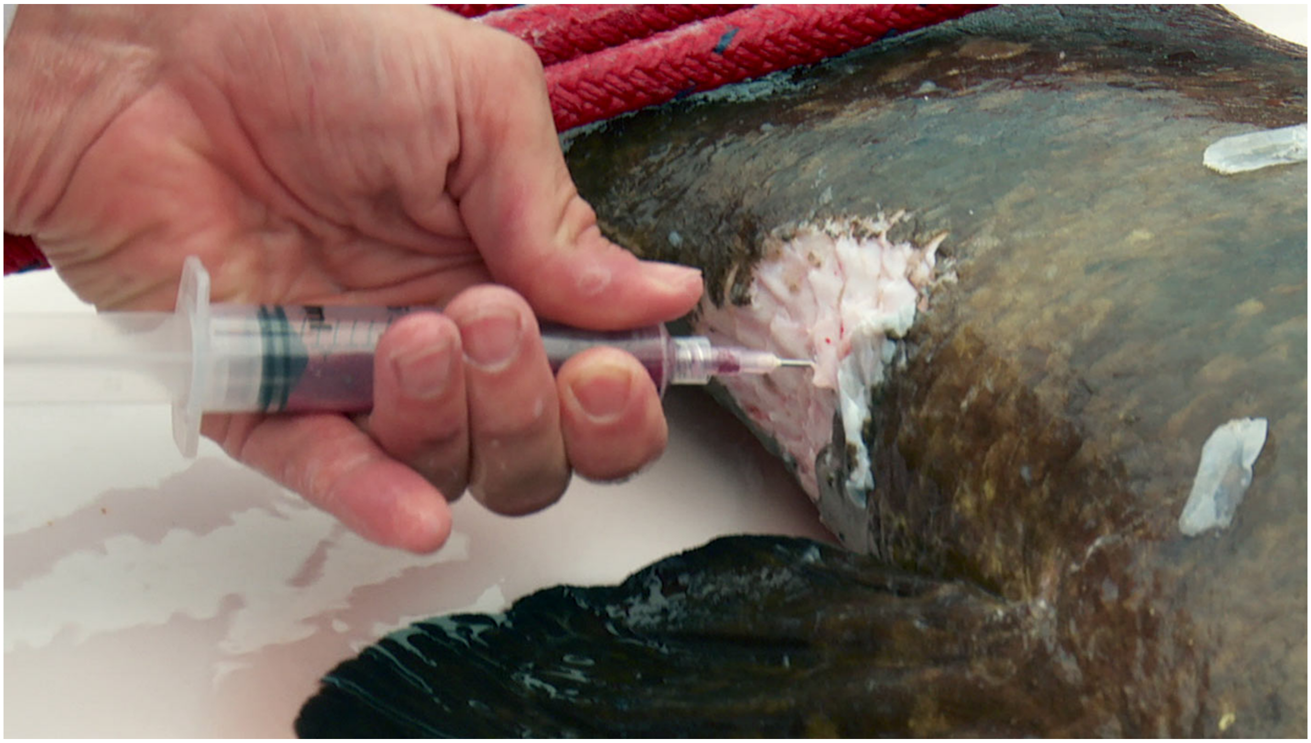

Supplemental Figure 1. Caudal venipuncture of Atlantic Goliath Grouper (*Epinephelus itajara*) using a sterile heparin-coated needle and syringe. Prior to puncture, scales were removed and the site was repeatedly prepared with sterile 70% isopropyl alcohol pads. Based on evidence from recaptured individuals, the area where scales were removed heals rapidly with new tissue growth.
