# Supplementary figures and images for: The Iconic Atlantic Goliath Grouper (Epinephelus itajara): A Comprehensive Assessment of Health Indices in the Southeastern United States Population
Source: Front Vet Sci. 2020 Sep 25;7:635. doi: 10.3389/fvets.2020.00635 (PMC7546827; doi:10.3389/fvets.2020.00635)

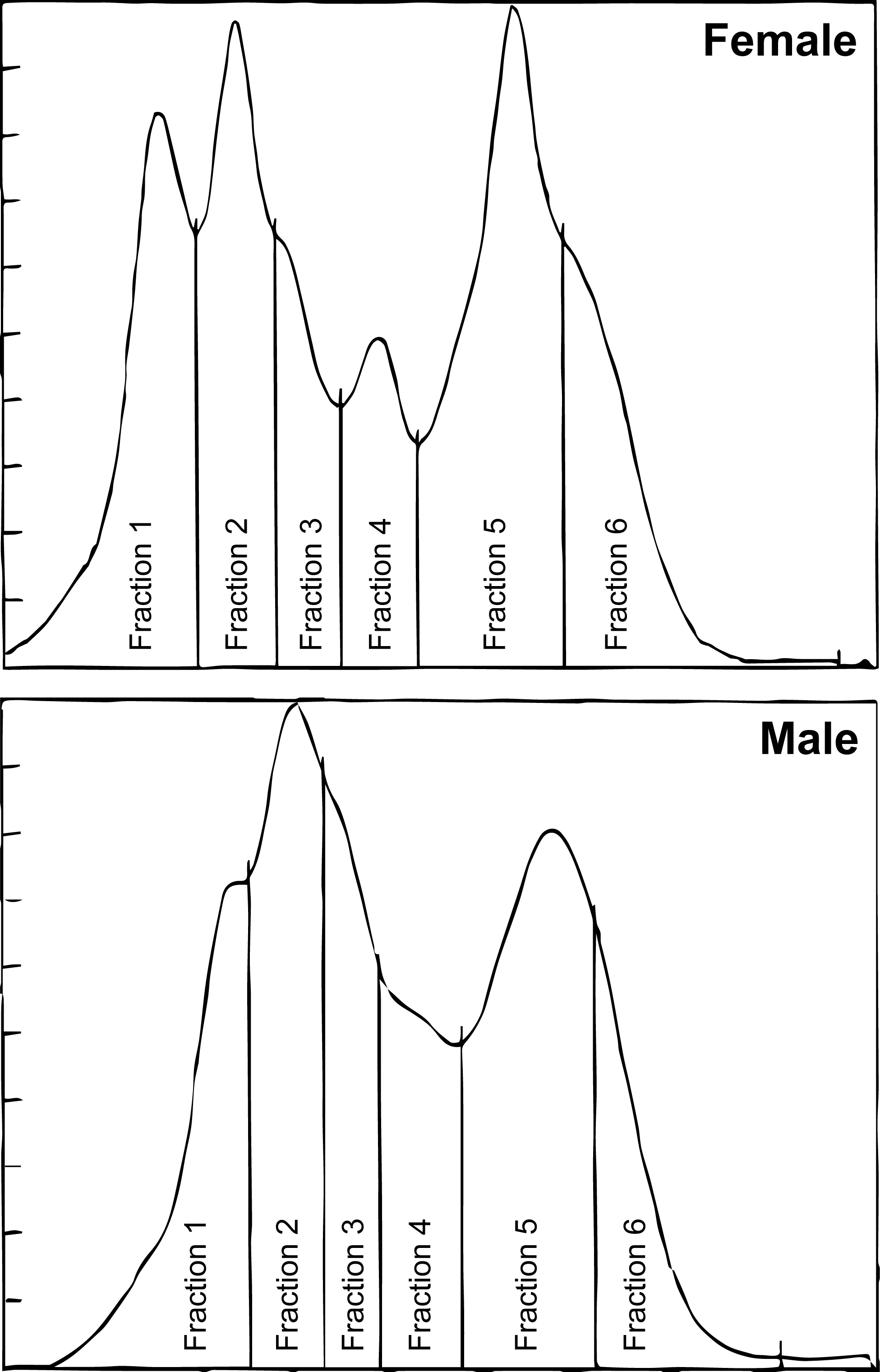

Supplement: Supplementary file 7 [file Image_2.tif]
