# Supplementary material for: The Iconic Atlantic Goliath Grouper (Epinephelus itajara): A Comprehensive Assessment of Health Indices in the Southeastern United States Population
Source: Front Vet Sci. 2020 Sep 25;7:635. doi: 10.3389/fvets.2020.00635 (PMC7546827; doi:10.3389/fvets.2020.00635)
Supplement: Supplementary file 8 [file Image_3.pdf]

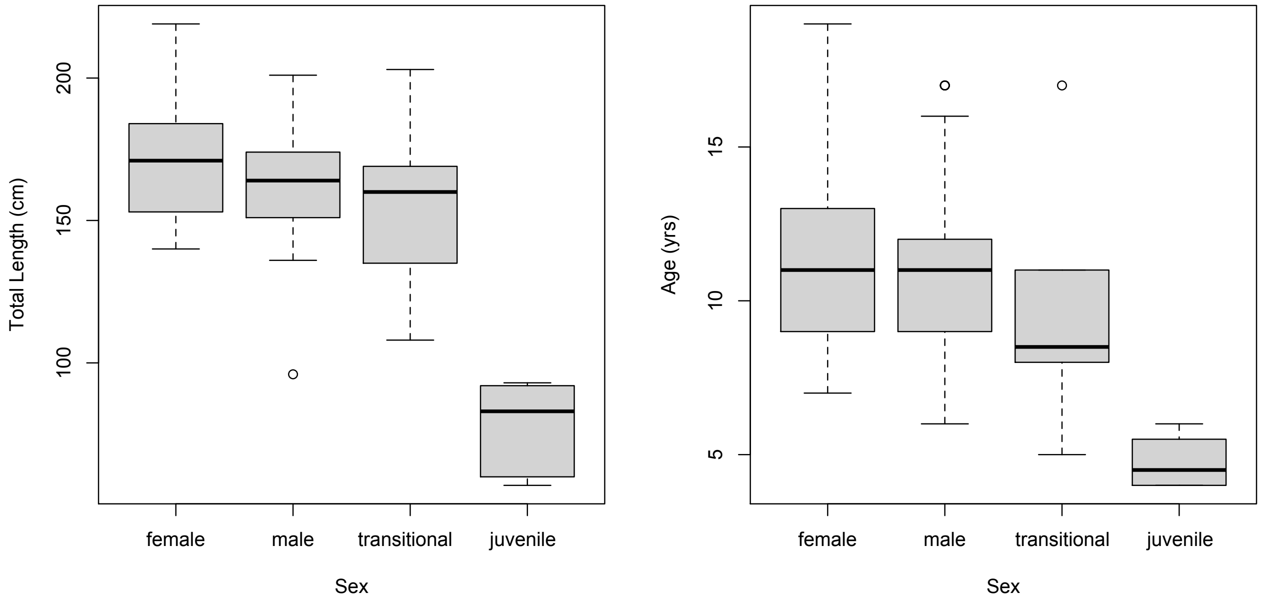

Supplemental Figure 3. Boxplots showing histologically determined sex categories (female, male, transitional) and juvenile age class by total length (cm) and age (yrs) for Atlantic Goliath Grouper (*Epinephelus itajara*) caught off Florida coasts. For total length (left figure), females were significantly ($p = 0.03$) larger than males. No other significant differences occurred between sexes, and juveniles were excluded from this analysis because we were only interested in comparing differences across mature subadults/adults. Range = vertical dashed lines, median = bold horizontal lines in box, first quartile = area below the line in the box, third quartile = area above the line in the box. The upper and lower ends of the nominal data range is defined as the respective interquartile distance (IQD) $\pm$ 1.5 IQD. Open circles = points that fall outside of this range.
